# Dietary methionine supplementation promotes mice hematopoiesis after irradiation

**DOI:** 10.1186/s40779-024-00584-x

**Published:** 2024-12-20

**Authors:** Wei-Wei Zhang, Yang Xiang, Li Chen, Shao-Ting Liu, Chuan-Chuan Lin, Jiu-Xuan Li, Li-Xin Xiang, Nan-Xi Chen, Dong-Ling Shi, Yang-Yang Zhang, Xue-Ying Wang, Lan-Yue Hu, Sai Chen, Ya Luo, Cheng-Ning Tan, Pei-Pei Xue, Yang-Zhou Jiang, Sheng-Wen Calvin Li, Zhen-Xing Yang, Ji-Gang Dai, Zhong-Jun Li, Qian Ran

**Affiliations:** 1 Department of Blood Transfusion, Laboratory Medicine Center, the Second Affiliated Hospital, Army Medical University, Chongqing, 400037 China; 2 Hematopoietic Acute Radiation Syndrome Medical and Pharmaceutical Basic Research Innovation Center, Ministry of Education of the People’s Republic of China, Chongqing, 400037 China; 3 Department of Nutrition, The Second Affiliated Hospital, Army Medical University, Chongqing, 400037 China; 4 Neuro-Oncology and Stem Cell Research Laboratory, CHOC Children’s Research Institute, Children’s Hospital of Orange County (CHOC), Orange, CA 92868-3874 USA; 5 Department of Neurology, University of California - Irvine School of Medicine, Orange, CA 92868 USA; 6 Department of Thoracic Surgery, the Second Affiliated Hospital, Army Medical University, Chongqing, 400037 China

**Keywords:** Irradiation, Methionine, Macrophage, S100A4, Bone marrow hematopoiesis

## Abstract

**Background:**

With the increasing risk of nuclear exposure, more attention has been paid to the prevention and treatment of acute radiation syndrome (ARS). Although amino acids are key nutrients involved in hematopoietic regulation, the impacts of amino acids on bone marrow hematopoiesis following irradiation and the associated mechanisms have not been fully elucidated. Hence, it is of paramount importance to study the changes in amino acid metabolism after irradiation and their effects on hematopoiesis as well as the related mechanisms.

**Methods:**

The content of serum amino acids was analyzed using metabolomic sequencing. The survival rate and body weight of the irradiated mice were detected after altering the methionine content in the diet. Extracellular matrix (ECM) protein analysis was performed via proteomics analysis. Inflammatory factors were examined by enzyme-linked immunosorbent assay (ELISA). Flow cytometry, Western blotting, and immunofluorescence were employed to determine the mechanism by which S100 calcium-binding protein A4 (S100A4) regulates macrophage polarization.

**Results:**

The survival time of irradiated mice was significantly associated with alterations in multiple amino acids, particularly methionine. A high methionine diet promoted irradiation tolerance, especially in the recovery of bone marrow hematopoiesis, yet with dose limitations. Folate metabolism could partially alleviate the dose bottleneck by reducing the accumulation of homocysteine. Mechanistically, high methionine levels maintained the abundance of ECM components, including collagens and glycoproteins, in the bone marrow post-irradiation, among which the level of S100A4 was significantly changed. S100A4 regulated macrophage polarization via the STAT3 pathway, inhibited bone marrow inflammation and facilitated the proliferation and differentiation of hematopoietic stem/progenitor cells.

**Conclusions:**

We have demonstrated that an appropriate elevation in dietary methionine enhances irradiation tolerance in mice and explains the mechanism by which methionine regulates bone marrow hematopoiesis after irradiation.

**Supplementary Information:**

The online version contains supplementary material available at 10.1186/s40779-024-00584-x.

## Background

With the rapid development of nuclear energy, nuclear medicine, and space technology, along with the escalating threat of nuclear war and nuclear terrorism, military personnel and civilians are exposed to the risk of damage from nuclear irradiation increasingly. Irradiation gives rise to a series of syndromes known as acute radiation syndrome (ARS) [1, 2]. The hematopoietic system is the most susceptible to irradiation [3]. Approximately 1–2 Gy can result in bone marrow hematopoietic suppression. Severe irradiation damage is able to cause bone marrow failure and immunodeficiency, further leading to hemorrhage, infection, multiple organ failure, and even death [4]. At present, the clinical treatment of irradiation-induced bone marrow hematopoietic injury is ineffective due to the difficulty of hematopoietic reconstitution. Therefore, exploring intervention strategies that facilitate the restoration of bone marrow hematopoiesis after irradiation is of great significance.

The repair of the structure and function of the hematopoietic niche is the key to bone marrow hematopoietic reconstitution. The hematopoietic niche primarily consists of cellular components, the extracellular matrix (ECM), and a cytokine network [5]. Many studies have shown that the ECM and cytokines regulate the homing, self-renewal, proliferation, and differentiation of hematopoietic stem cells (HSCs) [6–8]. Furthermore, alterations in amino acids are intricately related to the structure and functionality of the hematopoietic niche [9]. Under physiological conditions, the concentration of amino acids in bone marrow is 100-fold higher than that in peripheral blood [10]. At this time point, most HSCs are in a quiescent state, and the demand for energy metabolism is relatively low [11]. It has been reported that a variety of amino acid metabolisms can regulate HSC function. For example, valine deficiency inhibits HSC proliferation, and dietary serine alteration regulates ferroptosis susceptibility of HSCs [12–14]. Under stress conditions, such as irradiation, infection, or toxic stimulation, HSCs are activated, and the demand for protein increases when cells are in an activated state [15]. Glutamine can also modulate the activation of HSCs under inflammatory stress conditions [16]. The depletion of bone marrow cells was reduced in mice exposed to irradiation through the administration of arginine [17]. However, the metabolic changes of amino acids, their regulatory effects on hematopoiesis, and their influence on the bone marrow microenvironment after irradiation remain largely unexplored.

In this study, we used the mouse model with half-lethal dose (LD50) to investigate the characteristics of amino acid metabolism following irradiation. We screened out the key amino acids that regulate hematopoietic reconstitution in the bone marrow by analyzing the changes in amino acids. The study aims to provide a theoretical basis for the treatment strategy of hematopoietic reconstruction after radiation.

## Methods

### Mice

All the animal experiments were conducted using 8-week-old male C57BL/6 mice from Beijing Vital River Laboratory Animal Technology Co., Ltd., China. The mice were maintained in specific pathogen-free conditions at the Animal Experimental Center of the Army Medical University, with a humidity of (50 ± 5)%, a temperature of (24 ± 1) °C, and a 12 h light–dark cycle, and were provided with standard food and water. All procedures were approved by the Laboratory Animal Welfare and Ethics Committee of the Army Medical University (AMUWEC2020122, AMUWEC2020055, AMUWEC20211415, AMUWEC20224721) and carried out following the institutional guidelines. For total body irradiation, the mice received a single dose of 7 Gy ^60^Co γ-rays at a rate of 0.69 Gy/min.

A total of 739 C57BL/6 mice were randomly assigned to each group [*n* = 20 in the groups of the effect of dietary methionine change on mouse survival experiments, *n* = 10 in the groups of folate (FA) supplementation experiments, *n* = 3–6 in the other experiments as indicated in the figure legends]. In the control group, the mice were fed with a standard maintenance diet (AIN-93 M) [18]. The mice in the high methionine diet (H-Met) group were provided with an AIN-93 M diet supplemented with methionine (the final concentration of methionine was 10, 20, or 40 g/kg). The mice in the low methionine diet (L-Met) group were given an AIN-93 M diet with reduced methionine (0.2 g/kg) [19]. In the experiment of FA supplementation, the drinking water of the experimental group contained 20 mg/L of FA [20].

### Amino acid determination

After mice received irradiation, the survival rate and changes in body weight were recorded within 28 d. The mice were categorized into three groups based on their survival time: the early death (ED) group, the late death (LD) group, and the survival (S) group [21]. At 0, 3, 7, 14, 21, and 28 d after irradiation, approximately 80 μl of whole blood was collected from each mouse via the tail vein and immediately placed on ice. The samples were then centrifuged to extract the serum, which was subsequently stored at −80 °C. In each group, serum samples from 3 mice with similar body weights were pooled together (20 μl per mouse). After centrifugation, the supernatant was air-dried in liquid nitrogen and reconstituted for further analysis. Derivatization was performed before the supernatant was subjected to AB Sciex 4500MD liquid chromatography-tandem mass spectrometry for analysis. Amino acid quantification was conducted by comparing the standard and internal standard profiles, which are detailed in Additional file 1: Table S1.

### ECM protein determination

After irradiation, all mice were anesthetized using pentobarbital sodium (45 mg/kg). Then, the femurs were dissected and cleaned in sterile phosphate-buffered saline (PBS). Based on the CNMCS Compartmental Protein Extraction Kit (US Biological Life Sciences, C6012-25, USA), 100 mg of each femur was homogenized. Then, it was incubated in a series of buffers to sequentially remove the following proteins: cytosolic, nuclear, membrane, and cytoskeletal proteins. The insoluble pellet is the ECM-enriched fraction.

The ECM sample was dissolved in the lysate (9 mol/L urea, 50 mmol/L ammonium bicarbonate, and protease inhibitor) for protein extraction, followed by digestion, desalting, vacuum evaporation to dryness, and subsequent suspension in 0.1% formic acid water. An EASY-nLC 1200 system and a PepMap C18 25 cm × 75 μm × 1.7 μm column were used to separate the different components in the sample. The flow rate was 300 nl/min, with the mobile phase A consisting of 0.1% formic acid and the mobile phase B containing 0.1% formic acid and 80% acetonitrile. The program was as follows: 0.01–1 min, 4% B; 1–62 min, 4–24% B; 62–67 min, 24–40% B; 67–69 min, 40–100% B; and 69–75 min, 100% B.

An Orbitrap Explories 240 mass spectrometer (MS) was employed to analyze the mass of different components. The MS was operated in the positive electrode mode via nanoelectrospray ionization by heating the ion transfer tube, with the temperature set at 320 °C. MS analysis was conducted by acquiring MS1 scans within the range of 350–1500 m/z (R = 60,000), followed by a 2 s MS/MS scan acquisition, with an automatic gain control target value of 3 × 10^6^. MS2 spectra were obtained in the high-energy collision dissociation mode, with a normalized collision energy of 30%, a resolution of 17,500, and an isolation window of 1.6 m/z. The raw files were processed through Protein Discovery. A cut-off of a 1% false discovery rate was established at the peptide level.

### Extraction of peripheral blood

The mice were anesthetized with pentobarbital sodium. Blood was collected into tubes containing ethylene diamine tetraacetic acid (EDTA) using a microhematocrit tube, and bleeding was stopped by applying orbital pressure on the eye with a sterile cotton swab. A routine blood examination was performed via an automatic animal blood cell analyzer (Prandre XFA6030, Nanjing Prande Medical Equipment Co., Ltd., China) for peripheral blood count.

Regarding serum collection, the peripheral blood was collected into a centrifuge tube as described above, and the sample was placed at 4 °C for 2 h, Subsequently, the supernatant was collected by centrifugation (3000 rpm, 5 min).

### Flow cytometry

After the mice were treated with anesthetics and euthanized, the femurs and tibias were extracted. The contents of the bones were resuspended in 1 mg/ml collagenase IV buffer, and the crushed bone chips were incubated with 1 mg/ml of collagenase I buffer for 30 min at 37 °C. Both fractions were merged after the digestion step. Then, the precipitate was lysed with ACK lysis buffer (Beyotime, C3702, China) for 5 min. The cells were incubated with the corresponding flow cytometric antibodies for 30 min.

Regarding intracellular protein expression, the cells were initially stained for surface markers. After staining, the cells were immobilized with cell fixation (BioLegend, 420801, USA) and infiltrated with an osmotic solution (BioLegend, 421002, USA). Subsequently, the cells were incubated with intracellular protein antibodies for 30 min and with Alexa Fluor 488 for 30 min. Then, flow cytometry was carried out on a Gallios Flow Cytometer (Beckman, USA), and the data were analyzed through FlowJo V10 software (FlowJo LLC). The expression of inflammation factors was measured by adding brefeldin A (Invitrogen, 00-4506-51, USA) to the culture medium 3 h before cell collection.

The flow cytometry gating procedure is presented in Additional file 1: Table S2, and the antibody information is provided in Additional file 1: Table S3.

### Detection of EdU proportion

The Click-iT™ Plus EdU Flow Cytometry Assay Kits (Invitrogen, C10632, USA) were used to intraperitoneally inject the mice with 20 mg/kg EdU [22, 23]. The reaction was allowed to proceed for 1 h. Then, the bone marrow cells were harvested, and the cell surface markers were stained. The cells were fixed and stained with EdU. Finally, the cells were detected by flow cytometry. The antibodies information is provided in Additional file 1: Table S3.

### Immunofluorescence and hematoxylin and eosin (H&E) staining

After the mice were euthanized, the femurs and tibias were extracted and placed overnight in 4% paraformaldehyde at 4 °C. Subsequently, the femurs were placed in 10% EDTA (pH = 8) for 2 weeks, and incubated in 15% sucrose for 12 h, followed by another 12 h incubation in 30% sucrose. After that, the femurs were placed in optimal cutting temperature compound (OCT) medium and frozen at –80 °C until sectioning, 10 μm sections were cut at −20 °C using a freezing microtome and transferred to glass slides. After the sections were permeated and fixed, they were blocked for more than 2 h, incubated with antibodies at 4 °C overnight, and incubated with a secondary antibody for 2 h. The sections were imaged by confocal laser scanning microscopy. In addition, femurs were fixed, embedded in paraffin, then sectioned and stained with H&E. The information on the antibodies is provided in Additional file 1: Table S3.

### Isolation of bone marrow cells and supernatant

After the mice were anesthetized, femurs and tibias were collected and both ends of each bone were trimmed. A cannula was made by placing a 0.6 ml centrifuge tube with the bottom cut out in a 1.5 ml centrifuge tube. The bones were placed in a 0.6 ml microcentrifuge tube. After being centrifuged at 8000 rpm for 2 min, the bone marrow contents were collected in a 1.5 ml centrifuge tube [24].

### Enzyme-linked immunosorbent assay (ELISA)

S100 calcium-binding protein A4 (S100A4), interleukin (IL)-1β, tumor necrosis factor-α (TNF-α), IL-6, and IL-10 were measured using the mouse S100A4 ELISA Kit (JL20190), mouse IL-1β ELISA Kit (JL18442), mouse TNF-α ELISA Kit (JL10484), mouse IL-6 ELISA Kit (JL20268), and mouse IL-10 ELISA Kit (JL20242) (Jianglaibio, China) according to the manufacturers’ instructions. Briefly, the samples and standards were added to the sample wells and incubated at 37 °C for 1 h. Subsequently, the solution was discarded. Then, the detection antibody was added, and another incubation was performed at 37 °C for 1 h. The unbound antibody was then washed away, the substrate was added, and the mixture was incubated for 30 min. Finally, the termination solution was added, and the OD value was measured at a wavelength of 450 nm.

### Homocysteine determination

Homocysteine was determined using a Homocysteine (enzyme cycling method) content detection kit (Jianglaibio, JL-T1120, China) according to the manufacturer’s instructions. In brief, samples and standards were added to 96-well plates, followed by the addition of reagent 1. The mixture was then incubated at 37 °C for 5 min. Subsequently, reagent 2 was added and the solution was further incubated at 37 °C for another 2 min. The absorbance value A1 was measured at 340 nm and then measured again for 5 min as A2. Finally, the concentrations were calculated using the calculation method specified in the instruction manual.

### Cell culture and treatment

The RAW264.7 cells were obtained from the American Type Culture Collection (USA), and cultured in Dulbecco’s modified Eagle medium (Gibco, 11965092, USA), supplemented with 10% dialyzed fetal bovine serum (FBS; Thermo Fisher, 30067334, USA), 100 U/ml penicillin, and 0.1 mg/ml streptomycin (Thermo Fsher, 15070063, USA) at 37 °C in an atmosphere containing 5% CO_2_. To assess the effects of the methionine concentration in the medium on RAW264.7 cells, the modified Dulbecco’s modified Eagle medium with high glucose, no glutamine, no methionine, and no cystine (Gibco, 21013024, USA) was utilized. The medium was supplemented with 584 mg/L L-glutamine (Sigma, G7513, USA), 110 mg/L pyruvate (Gibco, 11360070, USA), 63 mg/L L-cystinodihydrochloride (Solarbio, C7480, China), and then methionine (Sigma, M5308, USA) was added as required (1.5, 3, 150, 300, 600, and 1200 mg/L). Normal Dulbecco’s modified Eagle medium with a methionine concentration of 30 mg/L was employed as a control.

To acquire bone marrow-derived macrophages (BMDMs), the tibias and femurs of the mice were isolated. Once both ends of the bones were removed, the bone marrow cells were resuspended using a sterile 1 ml syringe in sterile RPMI medium (Gibco, 31870082, USA) supplemented with 10% dialyzed FBS, 100 U/ml penicillin, and 0.1 mg/ml streptomycin. The cells were counted and inoculated in T75 cell culture flasks (1 × 10^7^ cells per flask) with 15 ml of RPMI medium containing mouse macrophage colony-stimulating factor (50 ng/ml; PeproTech, 315-02-100, USA), 10% dialyzed FBS, 100 U/ml penicillin, and 0.1 mg/ml streptomycin at 37 °C in an atmosphere containing 5% CO_2_. The medium was replaced on the fourth day. On day 7, the BMDMs were gently scraped off and inoculated into new culture flasks. To evaluate the effect of the methionine concentration in the medium on BMDMs, RPMI medium lacking methionine (Gibco, A1451701, USA) was used, and methionine was added as required (1.5, 3, 60, 150, 300, and 600 mg/L). A normal RPMI medium with a methionine concentration of 15 mg/L was utilized as a control.

In terms of irradiation therapy, the RAW264.7 cells and BMDMs were exposed to a single dose of 7 Gy of ^60^Co γ-rays at a rate of 0.69 Gy/min.

### Quantitative real-time PCR (qPCR)

Total RNA was extracted from cells using TRIzol reagent (TaKaRa, 9109, Japan). RNA purity and concentration were then examined via NanoDrop 2000. Subsequently, RNA was reversed transcribed into cDNA by the PrimeScript™ RT reagent Kit with gDNA Eraser (TaKaRa, RR047A, Japan). qPCR was conducted by TB Green^®^ Premix Ex Taq™ II (TaKaRa, RR820A, Japan). The reaction protocol was as follows: heating for 30 s at 95 °C, followed by 40 cycles of amplification (5 s at 95 °C and 30 s at 60 °C). The data were normalized relative to *β-actin.* The primer sequences are provided in Additional file 1: Table S4.

### Western blotting analysis

The cells were lysed in a lysis buffer (Beyotime, P0013B, China). The protein concentration was measured with a BCA protein quantification kit (Solarbio, PC0020, China). The protein samples were separated on SDS–polyacrylamide gels and transferred onto PVDF membranes. After being blocked with Western blocking buffer (Beyotime, P0023B, China), the membranes were incubated with the appropriate primary antibody overnight at 4 °C, and then with a horseradish peroxidase-conjugated secondary antibody. The blots were detected with enhanced chemiluminescence reagent (Absin, abs920, China), and images were acquired using a Photo-Image System (Fluor Quant AC600, AcuronBio, USA). The antibodies information is provided in Additional file 1: Table S3.

### Statistical analysis

All values are presented in the figures as the mean ± standard deviation. GraphPad Prism 9 software was employed for statistical analysis. The statistical significance of differences between the two groups was evaluated by a two-tailed Student’s *t*-test, the log-rank (Mantel-Cox) test, or the Mann–Whitney test. Results with a *P* < 0.05 were considered statistically significant.

## Results

### The survival time of irradiated mice is significantly associated with changes in multiple amino acids, particularly methionine

**Fig. 1:**
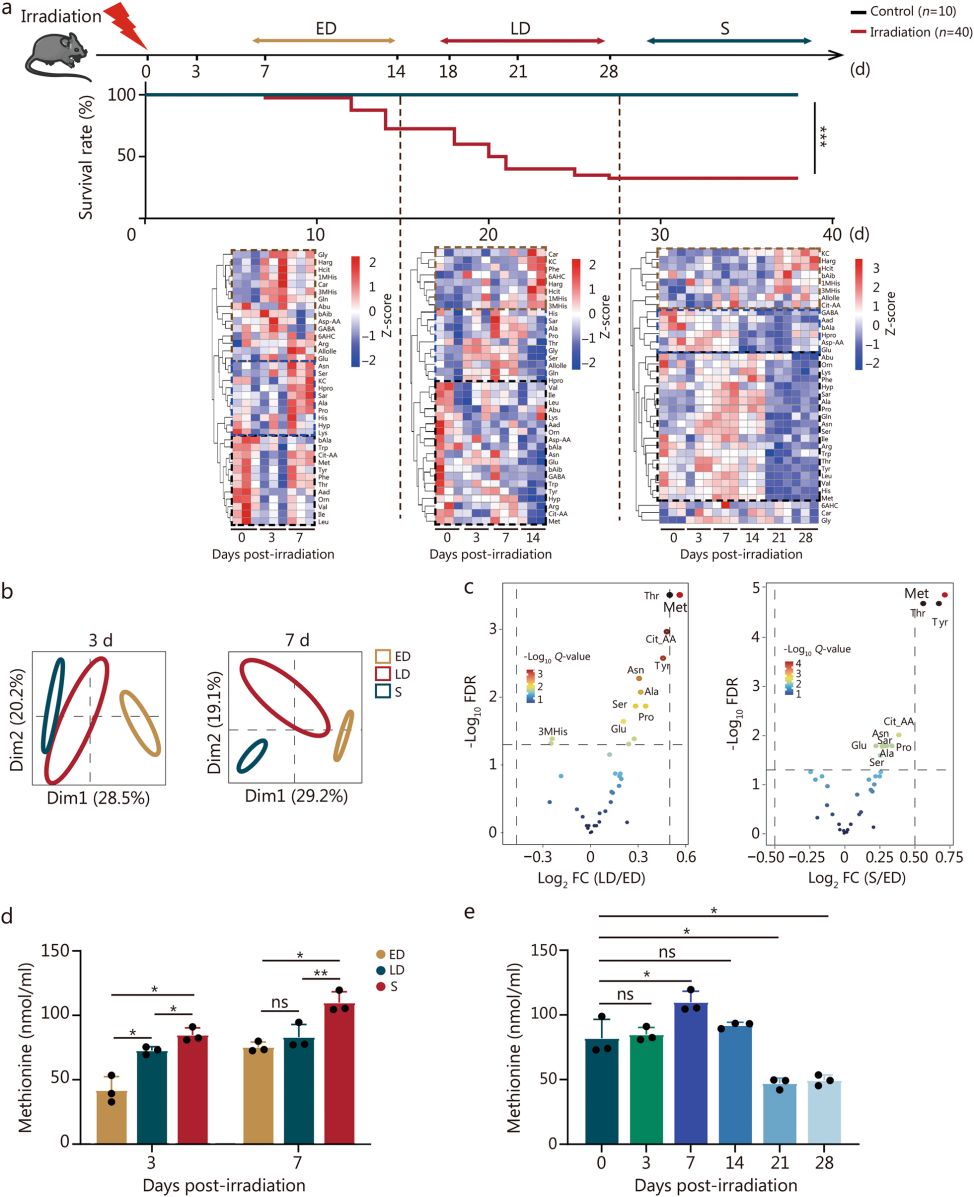
The survival time of irradiated mice is significantly correlated with changes in multiple amino acids, particularly methionine. **a** Schematic diagram of the experiment, survival rate of 8-week-old male C57BL/6 mice irradiated with 7 Gy (*n* = 10 in the control group and *n* = 40 in the IR group), and heatmap of the serum amino acid contents of the mice at different times of death after irradiation (*n* = 3). **b** Principal component analysis (PCA) plot of the three groups at 3 d or 7 d after irradiation. **c** Volcano plot depicting the significantly different amino acids among the three groups at 3 d after irradiation. **d** Changes in methionine content among the three groups at 3 d and 7 d after irradiation (*n* = 3). **e** Methionine content in the S group at different time points after irradiation (*n* = 3). The error bars indicate the standard deviation from three or more independent experimental replicates, ^*^P < 0.05, ^**^P < 0.01, ^***^P < 0.001, ns non-significant, as determined by the log-rank (Mantel-Cox) test (**a**) and Student’s *t*-test (**d**, **e**). ED early death, LD late death, S survival, FDR false discovery rate, Dim dimension, FC fold change, 1MHis 1-methylhistidine, 3MHis 3-methylhistidine, 6AHC 6-aminocaproic acid, Aad α-aminoadipate, Abu 2-aminobutyric acid, Ala alanine, Allolle valisoleucine, Arg arginine, Asn asparagine, Asp-AA aspartic acid, bAib 3-aminoisobutyric acid, bAla β-alanine, Car carnosine, Cit-AA citrulline, GABA γ-aminobutyric acid, Gln glutamine, Glu glutamic acid, Gly glycine, Harg homocysteine, Hcit homocitrulline, His histidine, Hpro homotypic proline, Hyp hydroxy-proline, Ile isoleucine, KC kynurenine, Leu leucine, Lys lysine, Met methionine, Orn ornithine, Phe phenylalanine, Pro proline, Sar sarcosine, Ser serine, Thr threonine, Trp tryptophan, Tyr tyrosine, Val valine

To explore the correlation between amino acid metabolism and irradiation survival in mice, serum samples were collected at diverse time points following LD50 irradiation. The mice exhibited two distinct mortality peaks, which occurred at 10–14 d and 18–21 d post-irradiation (Fig. 1a). Mice that died due to irradiation-induced mortality within the first peak were classified into the ED group, whereas those that passed away during the second peak were categorized into the LD group. The mice that survived for 30 d were placed in the S group. Our findings revealed differential amino acid profiles between deceased and surviving mice (Fig. 1a, b). The alterations of amino acid in each group at different time points after irradiation also varied (Additional file 1: Fig. S1a).

The patterns of amino acid changes varied among the distinct groups. In the ED group, the contents of methionine, valine, leucine, and other amino acids decreased at 3 d and increased at 7 d. Some amino acids like homoarginine increased at the early stage (3 d, 7 d) after irradiation, while γ-aminobutyric acid decreased on day 3 but then increased on day 7. In the LD group, the majority of amino acids such as methionine, valine, and leucine declined following irradiation, while tyrosine initially increased on days 3 and 7 but then decreased on day 14, and homoarginine specifically increased on day 14. In the S group, the majority of amino acids, including methionine, valine, and leucine, exhibited an initial increase from 3 to 14 d post-irradiation, followed by a subsequent decrease. Certain amino acids such as kynurenine and homoarginine, increased from 21 to 28 d after irradiation. A few amino acids, encompassing γ-aminobutyric acid and α-aminoadipic acid, continued to decline after irradiation (Fig. 1a). These findings indicate that the changes in various amino acid concentrations are associated with the survival time of mice after irradiation.

The volcano plot of 3 d post-irradiation suggested a significant difference in methionine among the three groups (Fig. 1c). Additionally, based on the changing trend of essential amino acids, only methionine was found to be significantly higher in the S group than in the death groups at 3 d and 7 d after irradiation (Fig. 1d, Additional file 1: Fig. S1b). Thus, we focused on methionine, which reached its peak at the highest level on day 7 after irradiation and subsequently declined (Fig. 1e).

### Dietary methionine supplementation promotes irradiation tolerance in mice, especially for hematopoiesis, but with dose limitations

**Fig. 2:**
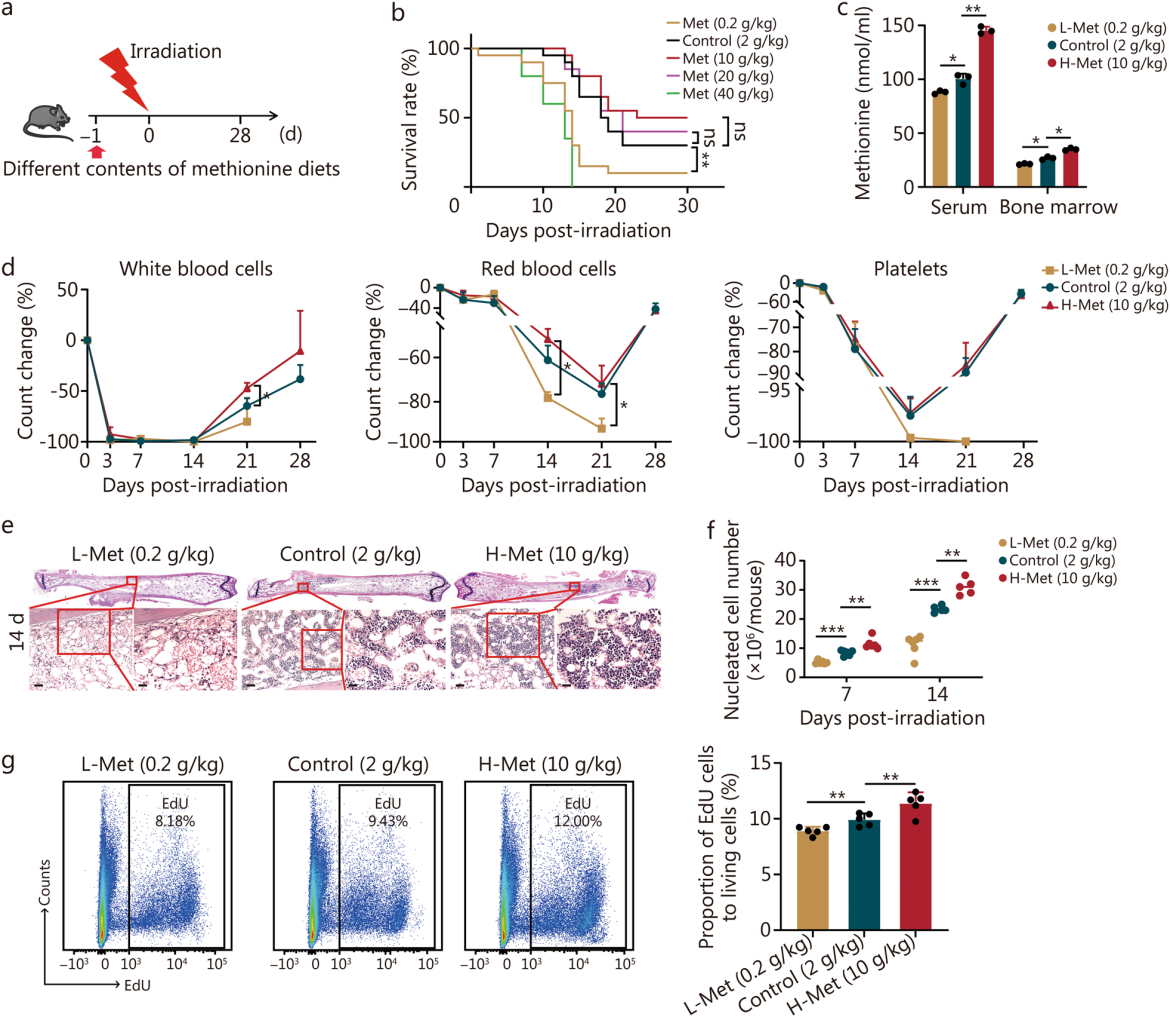
Dietary methionine supplementation enhances irradiation tolerance in mice, particularly for bone marrow hematopoiesis, but with dose limitation. **a** Schematic representation of the experiment, 8-week-old male C57BL/6 mice were irradiated with 7 Gy, and the mice were provided with diets supplemented with different concentrations of methionine after irradiation. **b** Survival rate of mice after irradiation by being fed with different methionine concentrations (*n* = 20). **c** Methionine content in the serum and bone marrow of mice fed with diets of different methionine concentrations at 7 d after 7 Gy irradiation (*n* = 3). **d** Changes in white blood cells, red blood cells, and platelets in the peripheral blood by routine analysis after 7 Gy irradiation (*n* = 6). **e** H&E staining of femurs at 14 d after irradiation with diets of different methionine contents. Scale bar = 50 μm (left)/20 μm (right). **f** The number of nucleated cells in bone marrow at 7 d and 14 d after irradiation with diets of different methionine contents (*n* = 5). **g** Bone marrow cells proliferation on day 7 after irradiation with diets of different methionine contents (*n* = 5). The error bars indicate the standard deviation from three or more independent experimental replicates, ^*^P < 0.05, ^**^P < 0.01, ^***^P < 0.001, ns non-significant, as determined by log-rank (Mantel-Cox) test (**b**) and Student’s *t*-test (**c, d, f, g**). Met methionine, L-Met low methionine diet, H-Met high methionine diet

To validate that methionine enhances the survival of mice after irradiation, we provided mice with diets having methionine concentrations of 0.2, 2, 10, 20, or 40 g/kg 1 d before irradiation (Fig. 2a). When the methionine concentration was lower than 2 g/kg (as the control), the survival rate was significantly reduced. While the methionine concentration increased to 10 g/kg, the survival rate improved. However, when the methionine concentration exceeded 10 g/kg, the survival rate started to decline (Fig. 2b). Notably, variations in the dietary methionine concentration resulted in changes in the food intake of the mice, with a decrease observed during the early irradiation period regardless of whether the methionine concentration was lower or higher than that of control. The body weights of the mice were also associated with the dietary methionine concentration, with the group receiving 10 g/kg methionine showing greater recovery than the other groups, whereas the group receiving 40 g/kg methionine experienced the most pronounced weight loss following irradiation exposure (Additional file 1: Fig. S2a). Therefore, dietary methionine affects the irradiation tolerance of mice but with dose limitations. For subsequent experiments, we adopted three concentration gradients: L-Met (0.2 g/kg), control (2 g/kg), and H-Met (10 g/kg).

We further validated that the intake of methionine at different concentrations directly influenced the serum and bone marrow methionine levels (Fig. 2c). The hematopoietic system is highly susceptible to irradiation [4]. A low methionine diet aggravated the irradiation damage to peripheral blood components, including white blood cells, red blood cells, and platelets (Fig. 2d). After irradiation, a high methionine diet facilitated the recovery of bone marrow nucleated cells, whereas a low methionine diet had the opposite effect (Fig. 2e, f; Additional file 1: Fig. S2b). Moreover, a high methionine diet increased the proportion of proliferating cells in bone marrow post-irradiation (Fig. 2g), similarly, the proportions of T cells, B cells, and myeloid cells increased on a high methionine diet (Additional file 1: Fig. S3).

In conclusion, a low methionine diet after irradiation augmented the vulnerability of the hematopoietic system of mice to damage and decreased their survival rate. Increasing the content of methionine in the diet improved the irradiation tolerance of the mice, particularly concerning hematopoiesis, but there was a dose limit.

### A high methionine diet produces excess homocysteine, which is partially rescued by FA supplementation

**Fig. 3:**
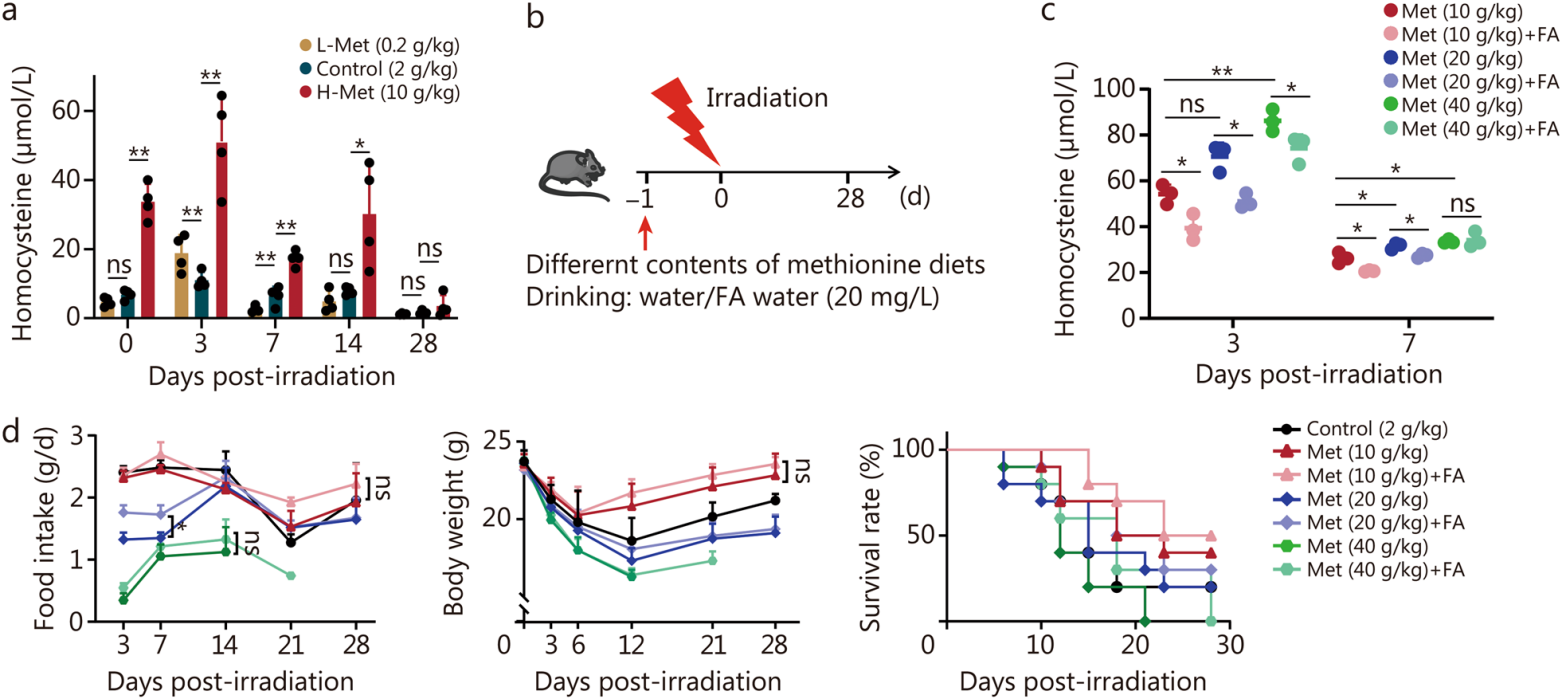
Folate (FA) supplementation can partially rescue the excess homocysteine produced by a high methionine diet. **a** Changes in the homocysteine content in the serum after irradiation (*n* = 4). **b** Schematic illustration of the experiment, 8-week-old male C57BL/6 mice were irradiated with 7 Gy, and the mice were provided with diets supplemented with different concentrations of methionine and FA in the water. **c** Serum homocysteine content of mice at 3 d and 7 d following FA supplementation after irradiation (*n* = 3). **d** Survival rate (*n* = 10), food intake (*n* = 3) and body weight (*n* = 10) of the mice after irradiation. The error bars indicate the standard deviation from three or more independent experimental replicates, ^*^P < 0.05, ^**^P < 0.01, ns non-significant, as determined by log-rank (Mantel-Cox) test (survival rate in** d**) and Student’s *t*-test (**a**, **c**, food intake and body weight in **d**). Met methionine, L-Met low methionine diet, H-Met high methionine diet

Methionine is involved in metabolism in vivo through the methionine cycle, of which homocysteine is a key metabolite [25–27]. On the one hand, methionine plays a pivotal role in glutathione production for regulating oxidative stress; on the other hand, methionine undergoes remethylation through the FA cycle to be reintegrated into the methionine cycle [28]. Notably, excessive accumulation of homocysteine led to hyperhomocysteinemia [29]. We found that the serum homocysteine content of mice increased significantly under a high methionine diet. Furthermore, the homocysteine concentration continued to rise as the dietary methionine concentration increased (Fig. 3a, c). Hence, it can be deduced that excessive accumulation of homocysteine may account for the dose-limiting effect of high methionine concentrations on irradiation tolerance in mice.

To investigate this hypothesis, we supplemented the drinking water of mice fed a high methionine diet with 20 mg/L FA (Fig. 3b). The results indicated that on day 3 post-irradiation, FA supplementation significantly alleviated the elevation in homocysteine levels induced by a high methionine diet, and FA effectively reduced the homocysteine content in both the 10 g/kg and 20 g/kg high methionine diets on day 7 post-irradiation (Fig. 3c). In the 10 g/kg methionine group, after FA supplementation, there was no significant increase in food intake and the body weights rose, while the mouse survival rate improved from 40 to 50%. In the 20 g/kg methionine group, after FA supplementation, the survival rate increased from 20 to 30%. In the 40 g/kg methionine group, FA supplementation extended the survival time of mice, there were no significant differences in food intake and body weight (Fig. 3d). Overall, FA supplementation can effectively mitigate the elevated homocysteine concentrations caused by a high methionine diet while enhancing mouse survival following irradiation exposure.

### Methionine maintained the abundance of the bone marrow ECM after irradiation, and S100A4 was significantly increased

The ECM, an essential component of the bone marrow microenvironment, is a highly dynamic structural network that undergoes continuous remodeling during both normal and pathological conditions [30, 31]. Traditionally regarded as an inert scaffold, it is now recognized as a dynamically interactive partner with the immune system and HSCs niche, especially in tumor research [6, 32–34]. Considering that the dietary methionine content affects irradiation tolerance in mice, and alterations in amino acid metabolism can significantly influence protein homeostasis, we hypothesized that dietary methionine might impact hematopoietic reconstitution through the ECM proteins post-irradiation. Then, we extracted ECM proteins from bones and characterized them via LC–MS/MS.

**Fig. 4:**
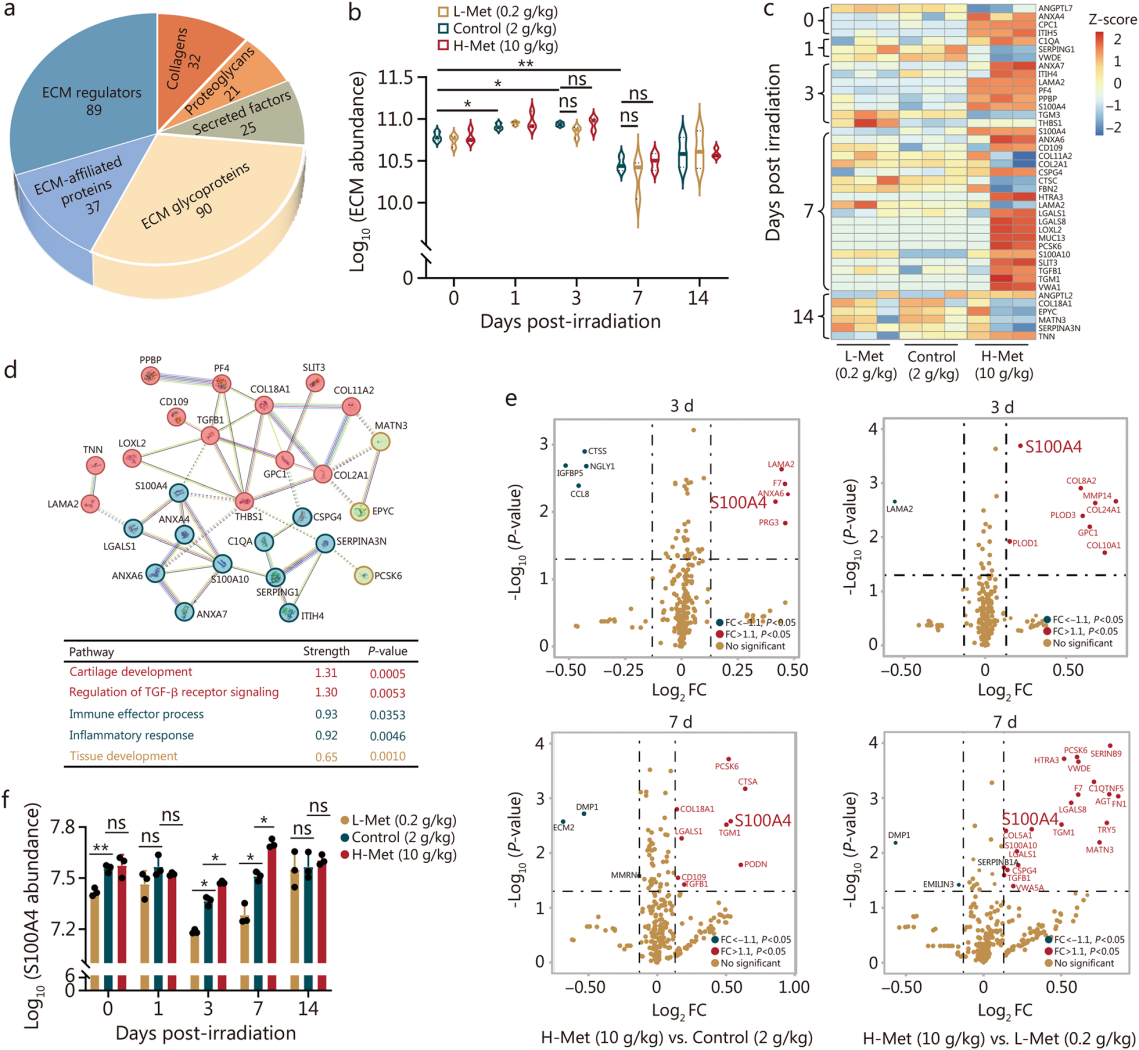
Methionine maintained the abundance of the bone marrow ECM after irradiation, and S100A4 was significantly elevated. **a** Pie chart of the number of proteins in all ECM categories. **b** The total abundance of ECM in the context of different methionine diets (*n* = 3). **c** Heatmap of differentially expressed proteins associated with different methionine diets (*n* = 3). **d** Protein–protein interaction (PPI) network of differentially expressed proteins associated with different methionine diets. **e** Volcano plot of the significantly different proteins associated with different methionine diets at 3 d and 7 d post-irradiation. **f** The abundance of S100A4 in response to different methionine diets (*n* = 3). The error bars indicate the standard deviation from three or more independent experimental replicates, ^*^P < 0.05, ^**^P < 0.01, ns non-significant, determined by Student’s *t*-test. ECM extracellular matrix, FC fold change, ANGPTL7 angiopoietin-like 7, ANGPTL2 angiopoietin-like 2, ANXA4 annexin A4, GPC1 glypican 1, ITIH5 inter-alpha (globulin) inhibitor H5, C1QA complement component 1Q subcomponent alpha polypeptide, SERPING1 serine (or cysteine) peptidase inhibitor clade G member 1, VWDE von Willebrand factor D and EGF domains, ANXA7 annexin A7, ITIH4 inter-alpha (globulin) inhibitor H4, LAMA2 laminin, alpha 2, PF4 platelet factor 4, PPBP pro-platelet basic protein, S100A4 S100 calcium binding protein A4, TGM3 transglutaminase 3, THBS1 thrombospondin 1, ANXA6 annexin A6, COL11A2 collagen type XI alpha 2, COL2A1 collagen type II alpha 1, CSPG4 chondroitin sulfate proteoglycan 4, CTSC cathepsin C, FBN2 fibrillin 2, HTRA3 HtrA serine peptidase 3, LGALS1 lectin galactose binding soluble 1, LGALS8 lectin galactose binding soluble 8, LOXL2 lysyl oxidase-like 2, MUC13 mucin 13, PCSK6 proprotein convertase subtilisin/kexin type 6, S100A10 S100 calcium binding protein A10, SLIT3 slit homolog 3, TGFB1 transforming growth factor β1, TGM1 transglutaminase 1, VWA1 von Willebrand factor A domain containing 1, COL18A1 collagen type XVIII alpha 1, EPYC epiphycan, MATN3 matrilin 3, SERPINA3N serine (or cysteine) peptidase inhibitor clade A member 3N, TNN tenascin N, CTSS cathepsin S, NGLY1 N-glycanase 1, IGFBP5 insulin-like growth factor binding protein 5, CCL8 chemokine (C–C motif) ligand 8, PRG3 proteoglycan 3, F7 coagulation factor VII, PLOD1 procollagen-lysine 2-oxoglutarate 5-dioxygenase 1, PLOD3 procollagen-lysine 2-oxoglutarate 5-dioxygenase 3, COL8A2 collagen type VIII alpha 2, COL24A1 collagen type XXIV alpha 1, MMP14 matrix metallopeptidase 14, COL10A1 collagen type XX alpha 1, DMP1 dentin matrix protein 1, EMILIN3 elastin microfibril interfacer 3, C1QTNF5 C1q and tumor necrosis factor related protein 5, AGT angiotensinogen, FN1 fibronectin 1, TRY5 trypsin 5, MMRN multimerin 1, ECM2 extracellular matrix protein 2, PODN podocan, CTSA cathepsin A, SERPINB9 serine (or cysteine) peptidase inhibitor clade B member 9, L-Met low methionine diet, H-Met high methionine diet

A total of 294 ECM proteins were identified (Fig. 4a). Principal component analysis (PCA) revealed significant differences in ECM among different time points post-irradiation (Additional file 1: Fig. S4a). ECM abundance was slightly increased at 1 d and 3 d, followed by a marked decrease at 7 d, gradual recovery at 14 d, and a high methionine diet promoted the increase of ECM abundance at 3 d and 7 d after irradiation, while there was no significant difference (Fig. 4b). The changes of collagens, ECM glycoproteins, proteoglycans, and ECM-affiliated proteins, were consistent with the total abundance results (Additional file 1: Fig. S4b). Notably, the largest number of differentially expressed proteins was detected on day 7 post-irradiation (Fig. 4c). The protein–protein interaction (PPI) network analysis unveiled significant enrichment for processes related to cartilage development, regulation of TGF-β receptor signaling, immune effector process, inflammatory response, and tissue development among these differentially expressed proteins (Fig. 4d).

A volcano plot disclosed significant variances in S100A4 expression among the three groups, and its involvement in inflammatory responses was predicted (Fig. 4d, e). S100A4, a member of the S100 calcium-binding protein family, is expressed in various cell types, including fibroblasts, macrophages, and tumor cells [35]. S100A4 expression was positively correlated with the dietary methionine concentration (Fig. 4f). Taken together, these results suggested that a high methionine diet regulated the ECM after irradiation, especially the abundance of S100A4, and that may contribute to irradiation resistance.

### A high methionine diet increased S100A4 expression in bone marrow macrophages and suppressed inflammation

**Fig. 5:**
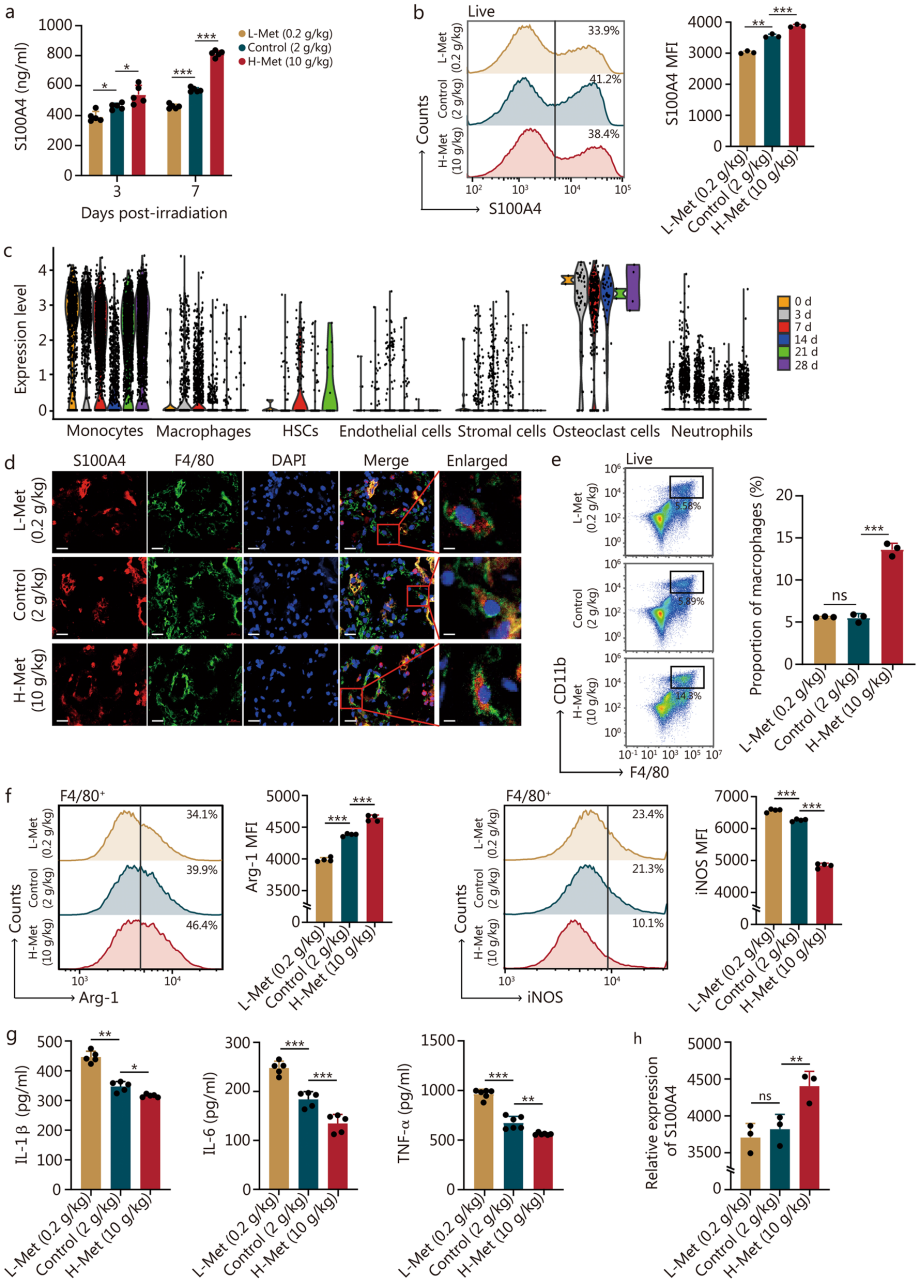
The high methionine diet enhanced S100A4 expression in bone marrow macrophages and suppressed inflammation. **a** The concentration of S100A4 in the bone marrow supernatant after irradiation with diverse methionine diets was determined via ELISA (*n* = 5). **b** The expression of S100A4 in bone marrow at 7 d after irradiation with different methionine diets was detected by flow cytometry (*n* = 3). **c** Transcriptional expression of S100A4 in bone marrow cells at various time points after irradiation. **d** Immunofluorescence results demonstrated that macrophages colocalized with S100A4. Scale bar = 20 μm. **e** Proportion of macrophages at 7 d after irradiation with different methionine diets (*n* = 3). **f** Expression of Arg-1 and iNOS in bone marrow macrophages at 7 d after irradiation in the setting of different methionine diets (*n* = 4). **g** Levels of inflammatory factors including IL-1β (*n* = 5), IL-6 (*n* = 5), and TNF-α (*n* = 6) in the bone marrow supernatant at 7 d of irradiation with different methionine concentrations. **h** The relative expression of S100A4 detected by transcriptome sequencing in macrophages at 7 d after irradiation with different methionine diets (*n* = 3). The error bars indicate the standard deviation from three or more independent experimental replicates, ^*^P < 0.05, ^**^P < 0.01, ^***^P < 0.001, ns non-significant, as determined by Student’s *t*-test. S100A4 S100 calcium-binding protein A4, MFI mean fluorescent intensity, HSCs hematopoietic stem cells, DAPI 4',6-diamidino-2-phenylindole, Arg-1 arginase 1, iNOS inducible nitric oxide synthase, TNF-α tumor necrosis factor-α, IL interleukin, L-Met low methionine diet, H-Met high methionine diet

The ELISA and flow cytometry results further confirmed that S100A4 protein levels in the bone marrow of mice were positively associated with dietary methionine concentrations at 3 d and 7 d post-irradiation (Fig. 5a, b). Analysis of S100A4 transcript levels in different bone marrow cell populations showed that S100A4 expression was increased in macrophages (Fig. 5c). Furthermore, the results of immunofluorescence and flow cytometry demonstrated that the proportion of macrophages in bone marrow nucleated cells within the H-Met group was greater than that in the other groups at 7 d after irradiation (Fig. 5d, e), and S100A4 was coexpressed with macrophages was observed. However, not all macrophages exhibited S100A4 expression (Fig. 5d). Macrophages play a crucial role in the immune system, and S100A4 is associated with macrophage polarization. Therefore, we postulated that the level of dietary methionine might regulate the polarization of bone marrow macrophages post-irradiation. To test this, we investigated the polarization of bone marrow macrophages in mice with different concentrations of methionine diets on day 7 after irradiation. The results indicated that a high methionine diet promoted M2 polarization of bone marrow macrophages and inhibited M1 polarization (Fig. 5f).

To explore the relationship between S100A4 expression and macrophage polarization following irradiation, we assessed S100A4 expression levels in bone marrow macrophages. A high methionine diet increased the proportion of S100A4-positive macrophages and the expression of S100A4 in macrophages (Additional file 1: Fig. S5a). Moreover, we found that approximately 80% of M2 macrophages expressed S100A4, and similarly, a high methionine diet promoted S100A4 expression in CD206-positive cells (Additional file 1: Fig. S5b). Macrophage polarization plays a crucial role in the regulation of inflammation in the bone marrow. The ELISA results demonstrated that a high methionine diet inhibited the secretion of IL-1β, IL-6, and TNF-α in the bone marrow following irradiation, whereas a low methionine diet had the opposite effect (Fig. 5g). These results suggested that a high methionine diet may promote S100A4 expression in macrophages, thereby participating in the regulation of bone marrow inflammation.

Given that the association between macrophage endocytosis and methionine has been reported [36], we thus questioned whether different concentrations of methionine affected the macrophage endocytosis post-irradiation. Consistent with the results in vivo, low methionine concentrations inhibited BMDM proliferation after irradiation, while high methionine concentrations promoted proliferation, with a dose limit. Based on the effect of methionine concentration changes on the proliferation of BMDMs after irradiation, three concentrations were selected for the further experiment (L-Met: 1.5 mg/L; control: 15 mg/L; H-Met: 150 mg/L) (Additional file 1: Fig. S5c). In the absence of irradiation, the endocytosis of BMDMs was weakened when the methionine concentration in the medium increased or decreased. However, high methionine enhanced endocytosis in BMDMs, whereas low methionine had the opposite effect after irradiation (Additional file 1: Methods and Fig. S5d, e). TGF-β1 is a positive regulator of macrophage endocytosis and given that human Jurkat cells were endocytosed, we employed mouse *TGF-β1* primers to detect *TGF-β1* expression, and the qPCR results indicated that methionine positively regulated *TGF-β1* mRNA levels after irradiation (Additional file 1: Fig. S5f). Macrophage endocytosis can promote macrophage polarization and regulate the inflammatory response, therefore, we simulated the polarity changes of BMDMs in their process of endocytosis and unendocytosis of apoptotic bone marrow cells in vitro. Flow cytometry results revealed that high methionine promoted the M2 polarization of BMDMs without endocytosis after irradiation, and the M2 polarization of BMDMs after endocytosis was exacerbated (Additional file 1: Fig. S5g), indicating that high methionine and endocytosis jointly promoted the M2 polarization of BMDMs post-irradiation.

To investigate the regulatory mechanism of methionine on macrophage polarization, bone marrow macrophages under different methionine diets on day 7 post-irradiation were sorted by flow cytometry and underwent transcriptome sequencing (Additional file 1: Methods). S100A4 transcription levels in macrophages were significantly higher in the H-Met group than in the other two groups (Fig. 5h). Gene set enrichment analysis (GSEA) revealed that the “cytokines and inflammatory response” and the “JAK-STAT signaling pathway” were activated in the H-Met group compared with the L-Met group, suggesting that methionine may regulate bone marrow inflammation by regulating the JAK-STAT signaling pathway (Additional file 1: Fig. S5h).

In conclusion, a high methionine diet increases the proportion of bone marrow macrophages, promotes their M2 polarization, and suppresses bone marrow inflammation after irradiation. These effects are attributed to the upregulation of S100A4 by methionine, which may regulate the JAK-STAT signaling pathway and enhance macrophage endocytosis.

### S100A4 regulates macrophage polarization to participate in the bone marrow inflammatory response via STAT3

To explore the regulatory role of S100A4 in macrophages during inflammation, RAW264.7 cells, and BMDMs were incubated with varying concentrations of methionine. S100A4 protein levels in RAW264.7 cells and BMDMs were increased after irradiation (Additional file 1: Fig. S6a). Decreasing the methionine concentration inhibited RAW264.7 cell proliferation, whereas increasing the methionine concentration enhanced RAW264.7 cell proliferation up to a certain dose limit, which is consistent with the vivo results. According to the impact of different methionine concentrations on the proliferation of RAW264.7 cells after irradiation, three typical concentrations were chosen for subsequent experiments (L-Met: 1.5 mg/L; control: 30 mg/L; H-Met: 300 mg/L) (Additional file 1: Fig. S6b). High methionine also inhibited the apoptosis of RAW264.7 cells after irradiation (Additional file 1: Fig. S6c). The expression of S100A4 positively correlated with the concentration of methionine in both RAW264.7 cells and BMDMs. Moreover, this effect was exacerbated by irradiation (Additional file 1: Fig. S6d, e).

**Fig. 6:**
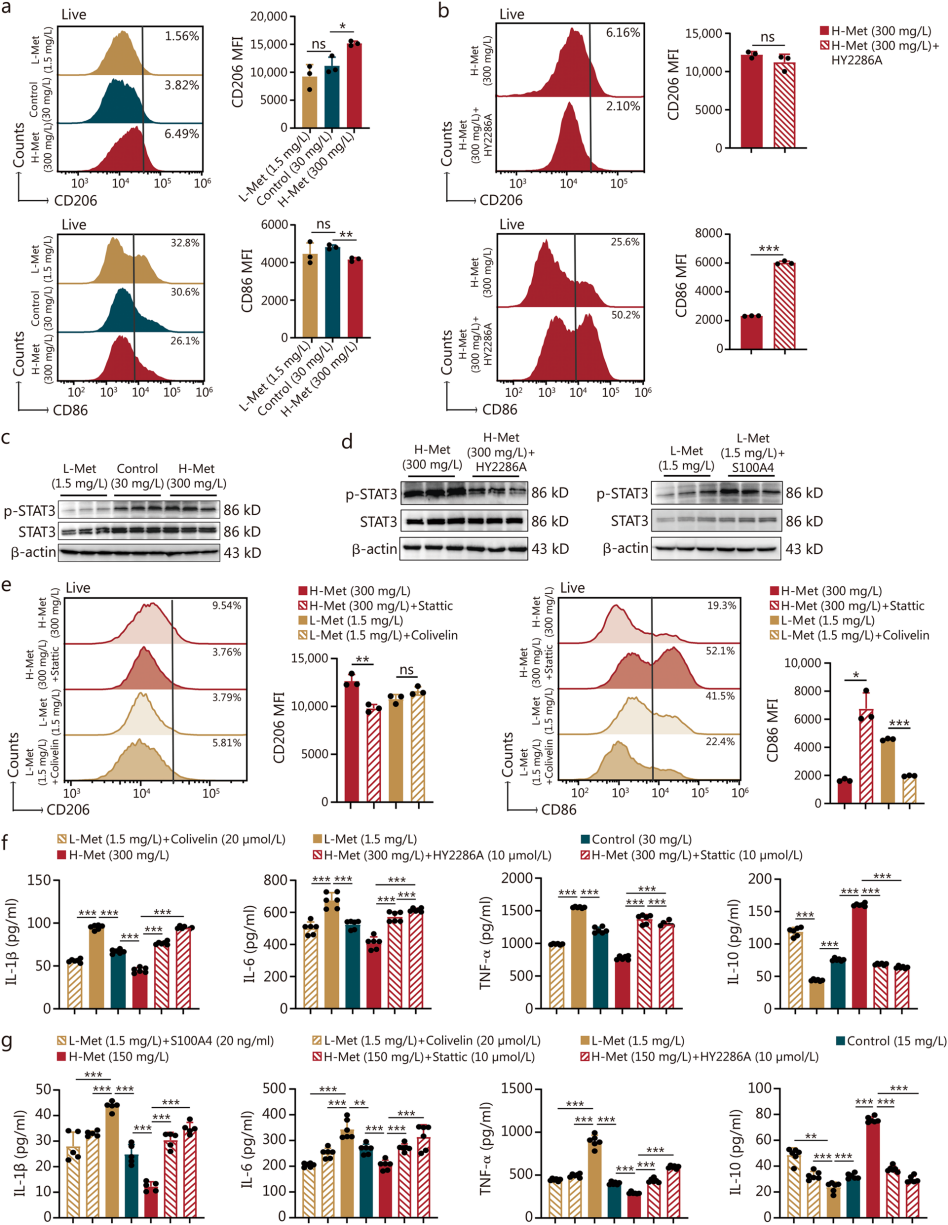
S100A4 regulates macrophage polarization to be involved in the bone marrow inflammatory response via STAT3. **a** The expression of CD206 and CD86 in RAW264.7 cells at 1 d after irradiation with different methionine media (*n* = 3). **b** The expression of CD206 and CD86 in RAW264.7 cells at 1 d after irradiation with the S100A4 inhibitor (HY2286A) in 300 mg/L methionine media (*n* = 3). **c** STAT3 protein expression was determined by Western blotting in RAW264.7 cells cultured in different methionine media. **d** STAT3 protein expression in RAW264.7 cells detected by Western blotting in the context of changes in S100A4 expression. **e** The expression of CD206 and CD86 in RAW264.7 cells at 1 d after irradiation with an STAT3 agonist (Colivelin) or inhibitor (Stattic) (*n* = 3). **f** The levels of inflammatory factors (IL-1β, IL-6, TNF-α, and IL-10) in the culture medium of RAW264.7 cells were measured via ELISA (*n* = 6). **g** The levels of inflammatory factors (IL-1β, IL-6, TNF-α, and IL-10) in the culture medium of BMDMs were measured via ELISA (*n* = 5). The error bars indicate the standard deviation from three or more independent experimental replicates, ^*^P < 0.05, ^**^P < 0.01, ^***^P < 0.001, ns non-significant, as determined by Student’s *t*-test. S100A4 S100 calcium-binding protein A4, MFI mean fluorescent intensity, TNF-α tumor necrosis factor-α, IL interleukin, L-Met low methionine diet, H-Met high methionine diet

Flow cytometry results revealed the expression of M1 macrophage marker CD86 and inducible nitric oxide synthase (iNOS) was inversely proportional to methionine concentration, while M2 macrophage marker CD206 was positively proportional to arginase-1 (Arg-1) (Fig. 6a, Additional file 1: Fig. S7a). To further investigate whether methionine regulates macrophage polarization through S100A4, a S100A4 inhibitor (HY2268A) was included in the culture medium of RAW264.7 cells. After irradiation, HY2286A promoted M1 polarization and inhibited M2 polarization in RAW264.7 cells (Fig. 6b, Additional file 1: Fig. S7b). Concurrently, we observed a reduction in the polarization of M1 and M2 macrophages upon *S100A4* knockdown, whereas *S100A4* overexpression led to an increase in polarization. However, the ratio of M1 to M2 significantly increased following *S100A4* knockdown and decreased after *S100A4* overexpression (Additional file 1: Methods and Fig. S7c-e) [37]. Thus, *S100A4* inhibition or knockdown in RAW264.7 cells promoted M1 polarization, while *S100A4* overexpression promoted M2 polarization.

Macrophage transcriptomics showed that a high methionine diet activated the JAK-STAT signaling pathway (Additional file 1: Fig. S5h). We confirmed that methionine and S100A4 regulate the STAT3 phosphorylation level (Fig. 6c, Additional file 1: Fig. S7f). HY2268A also inhibited the phosphorylation level of STAT3 (Fig. 6d). We observed that Colivelin, a STAT3 protein agonist, promotes M2 cell polarization while inhibiting M1 cell polarization, and the contrary effect was obtained with Stattic, a STAT3 inhibitor (Fig. 6e, Additional file 1: Fig. S7g, h).

Additionally, we quantified the secretion of inflammatory factors in RAW264.7 cells cultured in media supplemented with varying methionine concentrations by ELISA. Moreover, the high methionine medium was supplemented with a S100A4 inhibitor (HY2286A) and the STAT3 inhibitor (Stattic), while the low methionine medium was supplemented with the STAT3 agonist (Colivelin). The results indicated that high methionine levels increased the secretion of anti-inflammatory factor IL-10 while suppressing the secretion of pro-inflammatory factors (IL-1β, IL-6, and TNF-α). Conversely, the L-Met group showed opposite effects. Furthermore, the addition of HY2286A and Stattic significantly suppressed IL-10 secretion and promoted IL-1β, IL-6, and TNF-α secretion (Fig. 6f). In contrast, Colivelin exerted opposite effects. *S100A4* knockdown and overexpression also confirmed the effect of S100A4 on macrophage inflammation (Additional file 1: Fig. S7i). Moreover, we detected the levels of inflammatory factors secreted by BMDMs, which was consistent with the results obtained in RAW264.7 cells (Fig. 6g). Additionally, the results of flow cytometry and qPCR of inflammatory factors in RAW264.7 cells were in line with the ELISA results (Additional file 1: Fig. S8). These results suggested that methionine can regulate the secretion of inflammatory factors of macrophages via S100A4.

In conclusion, high methionine enhanced S100A4 expression in macrophages, promoting macrophage polarization and inhibiting the expression of inflammatory factors.

### Methionine promotes HSC/HSPC proliferation and differentiation by attenuating bone marrow inflammation

**Fig. 7:**
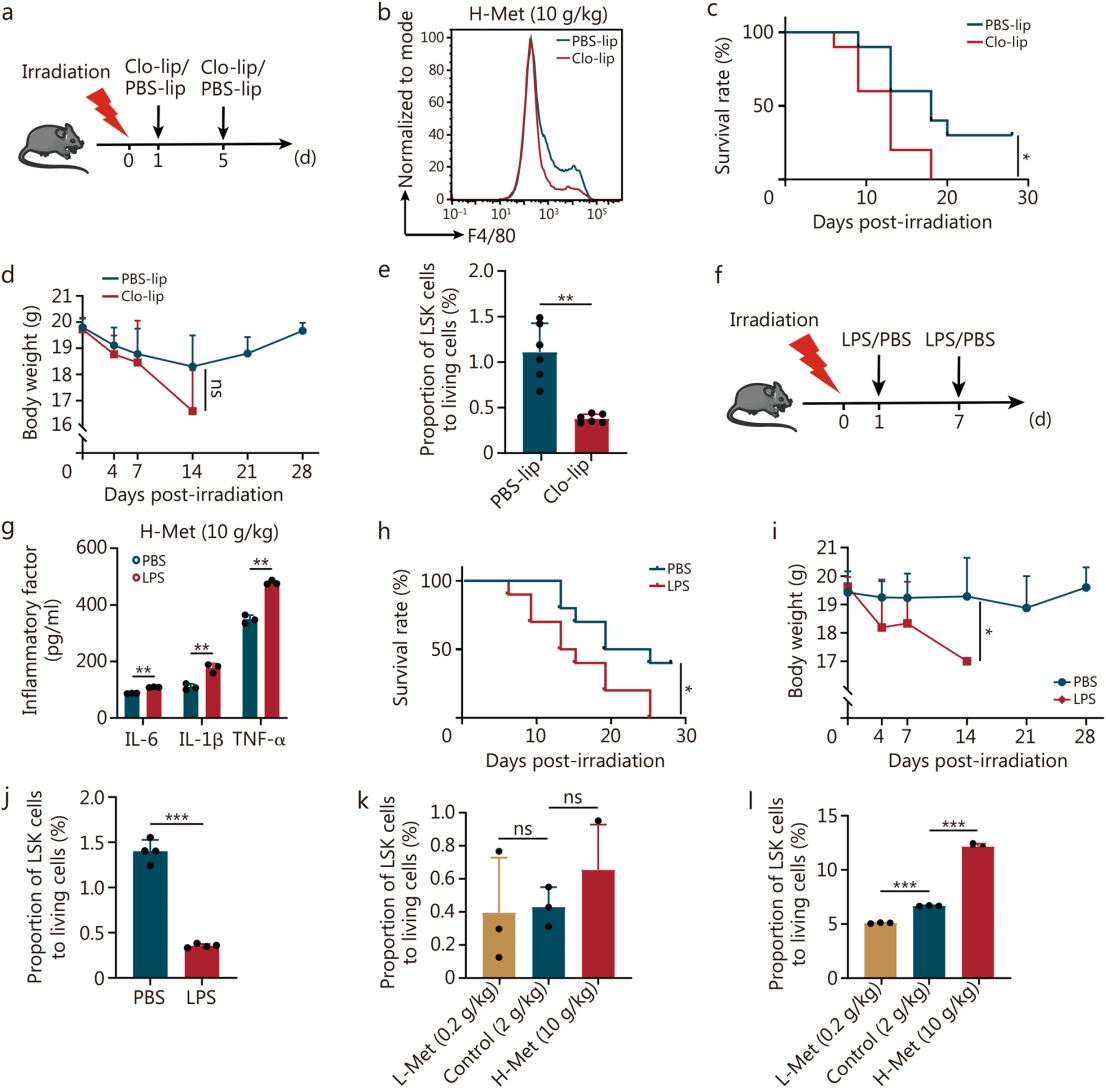
Methionine promotes HSC/HSPC proliferation and differentiation by alleviating bone marrow inflammation. **a** Schematic representation of the experiment with Clo-lip/PBS-lip; 8-week-old male C57BL/6 mice were irradiated with 7 Gy, and the mice were provided with diets supplemented with different concentrations of methionine and injected with Clo-lip/PBS-lip. **b** Flow cytometry was employed for the analysis of the expression of F4/80 (macrophage) in bone marrow with Clo-lip/PBS-lip on day 10 post-irradiation. Survival rates (**c**) and body weights (**d**) of the mice after irradiation with Clo-lip/PBS-lip (*n* = 10). **e** Proportion of LSK cells in the bone marrow of mice injected with Clo-lip/PBS-lip on day 10 post-irradiation (*n* = 6). **f** Schematic illustration of the experiment with LPS/PBS; 8-week-old C57BL/6 mice were irradiated with 7 Gy, and the mice were provided with diets supplemented with different concentrations of methionine and injected with LPS/PBS after irradiation. **g** ELISA was conducted for the determination of inflammatory factor levels in bone marrow supernatants after LPS/PBS injection on day 12 post-irradiation (*n* = 3). Survival rates (**h**) and body weights (**i**) of the mice after irradiation with LPS/PBS (*n* = 10).** j** Proportion of LSK cells in the bone marrow of mice injected with LPS/PBS on day 12 post-irradiation (*n* = 4). **k** Proportion of LSK cells in the bone marrow of mice fed different methionine diets on day 7 after irradiation (*n* = 3).** l** Proportion of LSK cells in the bone marrow of mice fed different methionine diets on day 14 after irradiation (*n* = 3). The error bars indicate the standard deviation from three or more independent experimental replicates, ^*^P < 0.05, ^**^P < 0.01, ^***^P < 0.001, ns non-significant, as determined by the log-rank (Mantel-Cox) test (**c, h**) and Student’s *t*-test (**d, e, g, i, j, k, l**). PBS phosphate buffer saline, Clo-lip clodronate liposomes, PBS-lip control liposomes, LPS lipopolysaccharide, HSC/HSPC hematopoietic stem/progenitor cell, TNF-α tumor necrosis factor-α, IL interleukin, L-Met low methionine diet, H-Met high methionine diet

To explore the role of macrophages in the promotion of hematopoietic reconstruction by methionine, we administered an intraperitoneal injection of Clo-lip (experimental group) or PBS-lip (control group) to irradiated mice fed a high methionine diet at 1 d and 5 d after irradiation (Fig. 7a). Depletion of macrophages significantly reduced the survival rate in the context of a high methionine diet post-irradiation, and the body weight of mice tended to decrease (Fig. 7b–d). The proportion of hematopoietic stem/progenitor cells (HSCs/HSPCs; LSK: Lineage^−^Sca-1^+^c-Kit^+^) cells was significantly reduced (Fig. 7e). Therefore, the regulation of macrophage by a high methionine diet plays an important role in the reconstitution of bone marrow hematopoiesis after irradiation.

To further investigate the potential of methionine in promoting hematopoietic reconstitution by regulating bone marrow inflammation, we administered an intraperitoneal injection of lipopolysaccharide (LPS) (experimental group)/PBS (control group) to irradiated mice fed a high methionine diet (Fig. 7f). LPS significantly stimulated the expression of inflammatory factors in the bone marrow supernatants (Fig. 7g). Following LPS injection, the survival rate and body weight were reduced (Fig. 7h, i). LPS injection significantly decreased the proportion of LSK cells (Fig. 7j).

Moreover, on day 7 post-irradiation, the dietary methionine concentration was positively correlated with the proportions of LSK cells, HSCs, and multipotential progenitor cells (MPPs), though not significantly (Fig. 7k, Additional file 1: Figs. S9a, S10a). However, the proportions of LSK cells, HSCs, and MPPs were significantly higher in the H-Met group at 14 d (Fig. 7l, Additional file 1: S10b). The proportion of proliferating LSK cells was larger than that in the other groups at 7 d but not significantly (Additional file 1: Fig. S10c). HSCs differentiate into common lymphoid progenitor cells (CLPs) and common myeloid progenitor cells (CMPs), and CMPs differentiate into granulocyte–macrophage progenitor cells (GMPs) and megakaryocyte-erythrocyte progenitor cells (MEPs). The proportion of CLPs, CMPs, and GMPs increased in the H-Met group compared with the control group at 7 d post-irradiation, but there was no significant difference in MEPs. (Additional file 1: Figs. S9b, c and S10d). Interestingly, a high methionine diet increased the proportion of CLPs without significant difference, while the proportions of CMPs, GMPs, and MEPs in the L-Met group were significantly higher than those in the control group at 14 d (Additional file 1: Fig. S10e).

## Discussion

**Fig. 8:**
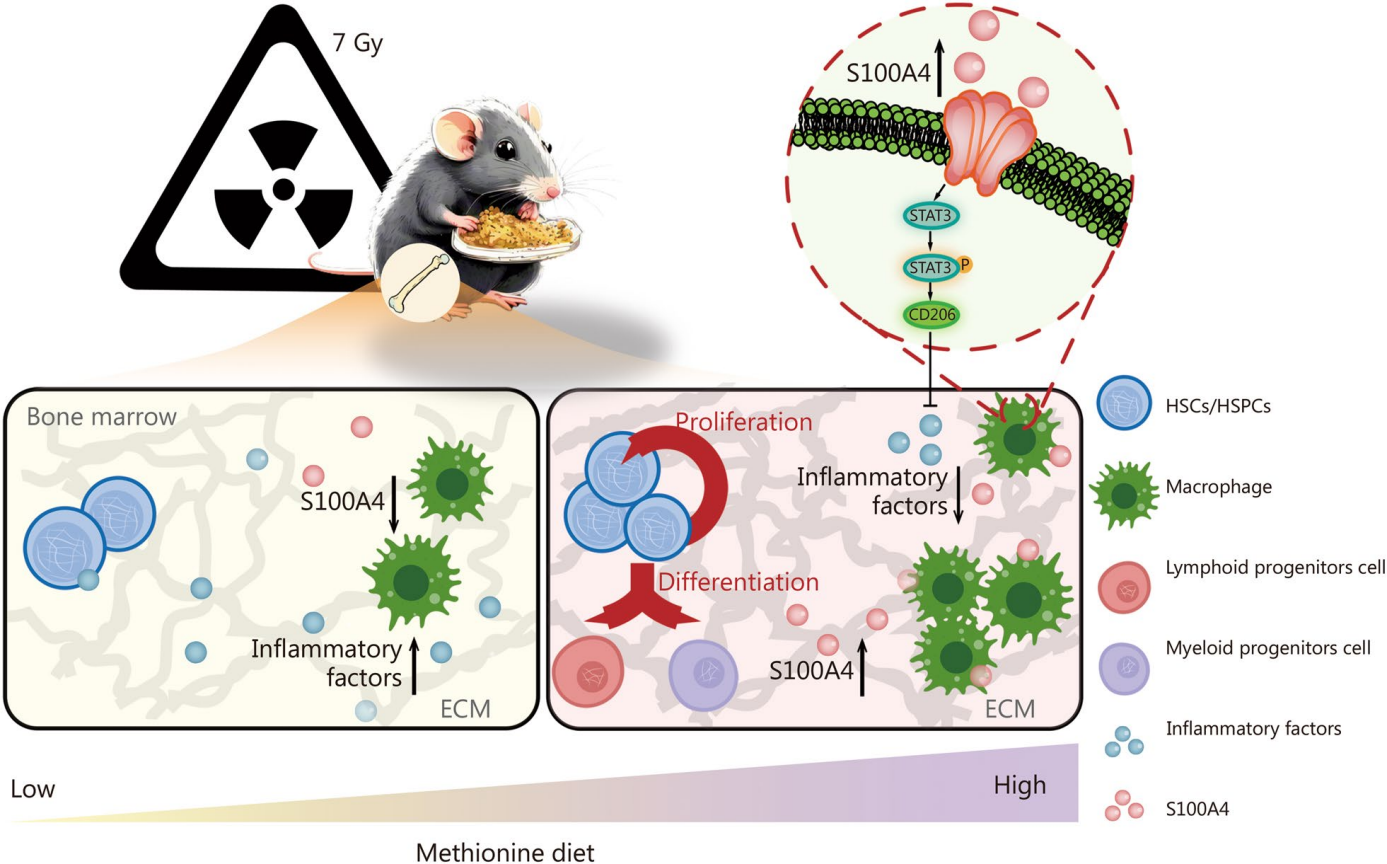
The mechanism by which dietary methionine supplementation promotes hematopoiesis in mice after irradiation. IR irradiation, HSCs/HSPCs: hematopoietic stem/progenitor cells, ECM extracellular matrix, S100A4 S100 calcium-binding protein A4

After irradiation, damage to the structure and function of the hematopoietic niche constitutes an important manifestation of pathological changes in the hematopoietic system. Although amino acids are recognized as crucial nutrients regulating the hematopoietic niche [10, 38, 39], there remains a considerable knowledge gap regarding the regulation of hematopoiesis by amino acid metabolism under stress conditions such as irradiation. In this study, through metabolomics analysis in a mouse model exposed to LD50 irradiation, we observed changes in the composition of various amino acids, especially methionine, that may be associated with irradiation tolerance in mice. Dietary methionine supplementation promotes post-irradiation survival and bone marrow hematopoietic reconstitution (Fig. 8).

Methionine plays a vital role in regulating oxidative stress through the generation of glutathione and gene methylation as a methyl donor and is thus proposed as a potential radiomitigator [28, 40, 41]. Our metabolomics results revealed that methionine is the amino acid with the most significant alteration in the serum of mice with diverse survival times following irradiation. Moreover, we observed that a dietary deficiency of methionine impeded bone marrow hematopoietic reconstitution, whereas an appropriate increase in methionine levels facilitated hematopoiesis. However, when the concentration of methionine surpasses 20 g/kg, it reduces the irradiation tolerance of mice, which may be attributed to the excessive accumulation of homocysteine. Methionine is highly toxic, and excessive methionine metabolism of it leads to hyperhomocysteinemia [42]. Additionally, dietary supplementation with methionine and FA in animals can partially alleviate the dose limitations by remethylating homocysteine back into methionine [43]. This discovery further confirms the hematopoietic reconstitution-promoting effect of methionine after irradiation. Furthermore, the metabolic changes in branched-chain amino acids and threonine were found to be positively correlated with mouse survival post-irradiation. The results are similar to those reported by other studies indicating that branched-chain amino acids can facilitate megakaryocyte differentiation and thrombopoiesis and that threonine can modulate oxidative stress and inflammation [13, 44, 45]. Therefore, these findings suggested that the changes in amino acid metabolism after irradiation could enhance the hematopoietic recovery and survival of mice.

A previous study has demonstrated that methionine can mediate gene methylation to regulate the proliferation and differentiation of stem cells and thereby modulate hematopoiesis in a leukemia mouse model [46]. In the present study, high methionine maintained the abundance of bone marrow ECM after irradiation, and the ECM protein S100A4 was significantly increased at 3 d and 7 d following irradiation. S100A4 is a paracrine and autocrine cytokine involved in the regulation of the inflammatory response. Our single-cell transcriptomic data revealed that the transcription level of S100A4 in macrophages was significantly higher than that in other cells, such as HSCs and endothelial cells, suggesting that macrophages may be the main cells secreting S100A4. Additionally, we found that high methionine levels post-irradiation facilitate M2 polarization and inhibit M1 polarization of macrophages. It has been documented that S100A4 promotes macrophage M2 polarization by modulating fatty acid oxidation [47]. The transcriptomic and in vitro experiments showed that S100A4 governs the STAT3 pathway to regulate macrophage M2 polarization. Furthermore, methionine modulates lipid metabolism and deposition [48], and lipid metabolism in macrophages also plays a crucial role in the regulation of inflammation [49]. We also ascertained that high methionine levels promote the expression of lipid metabolism-related genes (Additional file 1: Fig. S5i). These results imply that the augmented abundance of S100A4 induces M2 polarization of macrophages by regulating STAT3 phosphorylation post-irradiation. M1 macrophages possess a pro-inflammatory capacity, and M2 macrophages suppress inflammation and facilitate tissue repair [50]. Under stress conditions, the local inflammatory state of the bone marrow can directly modulate HSC/HSPC proliferation and differentiation [51]. We found that high methionine significantly decreased the levels of bone marrow inflammatory factors in vivo, and verified that the alleviation of bone marrow inflammation after irradiation promotes HSC/HSPC proliferation and differentiation as well as hematopoietic reconstitution.

This study also has certain limitations. First, a single dose of irradiation to mice at the LD50 is not sufficient to mimic the situation in patients with ARS. Additional doses of irradiation or small doses of prolonged irradiation are needed to further clarify the role of methionine. Second, this study demonstrated that methionine can regulate bone marrow inflammation through macrophage polarization. However, given that various cell types within the bone marrow can contribute to inflammation, further investigation is necessary to determine which cells, apart from macrophages, are involved in the methionine-mediated regulation of bone marrow inflammation. Third, although we confirmed that S100A4 is a key molecule in the regulation of macrophage polarization by methionine, it may not be the sole essential molecule involved; therefore, additional factors should be thoroughly examined to determine their contribution to methionine-induced regulation of macrophage polarization. Future studies should elucidate the complex underlying mechanisms involved. Currently, the treatment strategies for ARS include blood transfusions, anti-infective measures, and nutritional support [52]. However, owing to the limited patient population and the challenging long-term follow-up, there is still a lack of personalized and precise treatments for nutritional support [53]. Despite there are numerous limitations in our study, this study can offer a reference for the development of nutritional support treatment strategies.

## Conclusions

In this study, we initially demonstrated that an appropriate increase in dietary methionine enhances irradiation tolerance in mice, particularly facilitating bone marrow hematopoiesis. After irradiation, dietary methionine upregulated S100A4 expression in macrophages, mitigated the bone marrow inflammatory response, and promoted bone marrow hematopoiesis. These findings provide a theoretical foundation for interventions related to bone marrow hematopoietic reconstitution therapy after irradiation.

## Supplementary Information

**Additional file 1: Methods. Table S1** The standard and internal standard of amino acid determination. **Table S2** The gating of flow cytometry. **Table S3** The information of antibodies. **Table S4** The primer sequences. **Fig. S1** The changes of amino acid contents in the serum of mice at different time points post-irradiation. **Fig. S2** Dietary methionine supplementation promotes irradiation tolerance in mice. **Fig. S3** Dietary methionine supplementation facilitates the recovery of bone marrow cells. **Fig. S4** The expression of bone marrow extracellular matrix (ECM) with different methionine diets after irradiation. **Fig. S5** The high methionine diet increased S100A4 expression in bone marrow macrophages and promoted endocytosis. **Fig. S6** The effect of RAW264.7 cells and BMDMs cultured with different methionine mediums. **Fig. S7** S100A4 regulates macrophage polarization to participate in bone marrow inflammatory response via STAT3. **Fig. S8** The levels of inflammatory factors in RAW264.7 cells were detected via qPCR and flow cytometry. **Fig. S9** Flow cytometry analysis scheme for bone marrow cells. **Fig. S10** Methionine promotes the proliferation and differentiation of HSC/HSPC at 7 d and 14 d post-irradiation.

## Data Availability

Not applicable.

## Abbreviations

Arg-1: Arginase-1
ARS: Acute radiation syndrome
BMDMs: Bone marrow-derived macrophages
CMPs: Common myeloid progenitor cells
CLPs: Common lymphoid progenitor cells
ECM: Extracellular matrix
EDTA: Ethylene diamine tetraacetic acid
ELISA: Enzyme-linked immunosorbent assay
FA: Folate
FBS: Fetal bovine serum
GMPs: Granulocyte–macrophage progenitor cells
HSCs: Hematopoietic stem cells
HSCs/HSPCs: Hematopoietic stem/progenitor cells
IL: Interleukin
iNOS: Inducible nitric oxide synthase
LD50: Median lethal dose
LPS: Lipopolysaccharide
MEPs: Megakaryocyte-erythrocyte progenitor cells
MPPs: Multipotential progenitor cells
PCA: Principal component analysis
PPI: Protein–protein interaction
PBS: Phosphate-buffered saline
LSK: Lineage^−^Sca-1^+^c-Kit^+^
qPCR: Quantitative real-time PCR
S100A4: S100 calcium-binding protein A4
TNF-α: Tumor necrosis factor-α
