# Supplementary material for: Dietary methionine supplementation promotes mice hematopoiesis after irradiation
Source: Mil Med Res. 2024 Dec 20;11:83. doi: 10.1186/s40779-024-00584-x (PMC11660814; doi:10.1186/s40779-024-00584-x)
Supplement: Supplementary file 1 — Additional file 1: Methods. Table S1 The standard and internal standard of amino acid determination. Table S2 The gating of flow cytometry. Table S3 The information of antibodies. Table S4 The primer sequences. Fig. S1 The changes of amino acid contents in the serum of mice at different time points post-irradiation. Fig. S2 Dietary methionine supplementation promotes irradiation tolerance in mice. Fig. S3 Dietary methionine supplementation facilitates the recovery of bone marrow cells. Fig. S4 The expression of bone marrow extracellular matrix (ECM) with different methionine diets after irradiation. Fig. S5 The high methionine diet increased S100A4 expression in bone marrow macrophages and promoted endocytosis. Fig. S6 The effect of RAW264.7 cells and BMDMs cultured with different methionine mediums. Fig. S7 S100A4 regulates macrophage polarization to participate in bone marrow inflammatory response via STAT3. Fig. S8 The levels of inflammatory factors in RAW264.7 cells were detected via qPCR and flow cytometry. Fig. S9 Flow cytometry analysis scheme for bone marrow cells. Fig. S10 Methionine promotes the proliferation and differentiation of HSC/HSPC at 7 d and 14 d post-irradiation. [file 40779_2024_584_MOESM1_ESM.pdf]

Methods

Macrophage efferocytosis assay

BMDMs were plated in cell flasks and confocal dishes. Apoptotic cells (ACs) were represented by Jurkat cells exposed to 254 nm UV light for 30 min or bone marrow cells exposed to 7 Gy ^{60}Co γ-rays. ACs were labeled with PKH26 (Sigma, MIDI26-1KIT, USA) or PKH67 (Sigma, MIDI67-1KIT, USA) and then incubated at a 5:1 ratio (ACs:BMDMs). After 45 min, the unbound ACs were washed away, the cells were imaged through confocal laser scanning microscopy, and the cells in the culture flasks were collected for flow cytometry.

Cell sorting and RNA sequencing

Cell sorting was performed using a Cytoflex SRT cell sorter (Beckman, USA). F4/80^{+} cells were isolated from mouse bone marrow at 7 d after irradiation. The RNA of the sorted cells was extracted with a TRIzol reagent. RNA purity and concentration were examined via the NanoDrop 2000. RNA integrity and quantity were measured by the Agilent 2100/4200 system. After library preparation and pooling of diverse samples, the samples were subjected to Illumina sequencing (Berry Genomics, China). Raw data (raw reads) in FASTQ format were initially processed via in-house perl scripts. EdgeR was employed for differential expression analysis. The resulting *P*-values were adjusted using Benjamini and Hochberg's approach to control the false discovery rate. Genes with a $|\log_2$ fold change (FC)$| > 1$ and a *Q*-value < 0.05 were considered differentially expressed. All identified differentially expressed genes (DEGs) were subjected to Gene Ontology (GO) and functional enrichment analyses.

Cell transfection

For knockdown and overexpression of *S100A4* in RAW264.7 cells, the shRNA and overexpression plasmid targeting S100A4 was used to transfect into RAW264.7 cells. The shS100A4 and S100A4 overexpression plasmid (S100A4-pcdna3.1-egfp) was purchased from Shanghai Wei Huan Biological Technology Co. Ltd., China. The transfection reagent was Lipofectamine 3000 (Thermo Fisher, L3000015, USA), and the transfection steps were performed according to the manufacturer's protocol. The sequences of S100A4 siRNA were: shS100A4-1 forward 5'-CCACAAAUACUCAGGCAAATT-3', reverse 5'-UUUGCCUGAGUAUUUGUGGTT-3';

shS100A4-2 forward 5’-UCCAGAAGGUGAUGAGCAATT-3’, reverse 5’-UUGCUCAUCACCUUCUGGATT-3’; shS100A4-3 forward 5’-CAACAGGGACAAUGAAGUUTT-3’, reverse 5’-AACUUCAUUGUCCCUGUUGTT-3’.

Table S1 The standard and internal standard of amino acid determination

Reagent	Source
Internal standard of amino acid	1.2 ml, ZCI-MSK-A2, Cambridge Isotope Labratories, Inc.
D-proline	100 g, V900649, Sigma
L-glutamic acid	100 g, V900408, Sigma
Glycine	100 g, G7126, Sigma
D-leucine	2.5 g, 855448, Sigma
L-lysine	1 g, L5501, Sigma
L-hydroxyproline	25 g, V900395, Sigma
D-serine	5 g, S4250, Sigma
D-valine	5 g, 855987, Sigma
L-citrulline	1 g, C7629, Sigma
L-tyrosine	50 g, T3754, Sigma
L-histidine	10 g, H8000, Sigma
1-methyl-L-histidine	50 mg, 67520, Sigma
3-methyl-L-histidine	100 mg, M9005, Sigma
γ-aminobutyric acid	10 g, A5835, Sigma
L-aspartate	25 g, A8949, Sigma
L-asparagine	25 g, A0884, Sigma
L-threonine	1 g, T8625, Sigma
L-methionine	5 g, M9625, Sigma
D-kynurenine	25 mg, K2380, Sigma
L-ornithine monohydrochloride	100 g, V900374, Sigma
L-arginine	100 g, V900343, Sigma
DL-phenylalanine	100 g, V900615, Sigma
6-aminocaproic acid	25 g, A2504, Sigma
DL-2-aminoadipic acid	1 g, A0637, Sigma
DL-tryptophan	5 g, T3300, Sigma
D-alanine	5 g, A7377, Sigma

Sarcosine	100 g, 131776, Sigma
DL-3-aminoisobutyric acid	1 g, 217794, Sigma
L-homoarginine hydrochloride	5 g, H1007, Sigma
High proline	25 g, P45850, Sigma
D-2-aminobutyric acid	1 g, 116122, Sigma
β-alanine	25 g, 146064, Sigma

Table S2 The gating of flow cytometry

**Cells type**	**Flow cytometry gating**
Macrophages	F4/80^{+}CD11b^{+}
CD206^{+} macrophages	F4/80^{+}CD11b^{+}CD206^{+}
Arg-1^{+} macrophages	F4/80^{+}CD11b^{+}Arg-1^{+}
iNOS^{+} macrophages	F4/80^{+}CD11b^{+}iNOS^{+}
LSK cells	Lineage^{-}Sca-1^{-}c-Kit^{+}
HSCs	Lineage^{-}Sca-1^{-}c-Kit^{+}CD48^{-}CD150^{+}
MPPs	Lineage^{-}Sca-1^{-}c-Kit^{+}CD48^{-}CD150^{-}
CLPs	Lineage^{-}CD127^{+}Sca-1^{low}c-Kit^{low}
CMPs	Lineage^{-}Sca1^{-}c-Kit^{+}CD34^{+}CD16/32^{-}
GMPs	Lineage^{-}Sca1^{-}c-Kit^{+}CD34^{+}CD16/32^{+}
MEPs	Lineage^{-}Sca1^{-}c-Kit^{+}CD34^{-}CD16/32^{-}
T cells	CD4^{+}CD8a^{+}
B cells	CD19^{+}
Myeloid cells	CD19^{-}CD4^{-}CD8a^{-}NK1.1^{-}CD11b^{+}

iNOS inducible nitric oxide synthase, *LSK cells* hematopoietic stem/progenitor cells (HSCs/HSPCs), *HSCs* hematopoietic stem cells, *MPPs* multipotential progenitor cells, *CLPs* common lymphoid progenitor cells, *CMPs* common myeloid progenitor cells, *GMPs* granulocyte-macrophage progenitor cells, *MEPs* megakaryocyte-erythrocyte progenitor cells

Table S3 The information of antibodies

Reagent or resource	Source	Dilution
APC-Cy7 CD11b	101226, BioLegend, USA	1:100
APC F4/80	123116, BioLegend, USA	1:100
PE CD206	141706, BioLegend, USA	1:200
AF700 Lineage	133313, BioLegend, USA	1:100
PE Sca-1	108108, BioLegend, USA	1:100
PE-Cy5 c-Kit	105810, BioLegend, USA	1:100
PE-Cy7 CD34	119326, BioLegend, USA	1:100
APC-Cy7 CD127	135040, BioLegend, USA	1:100
BV421 CD16/32	101332, BioLegend, USA	1:100
APC-Cy7 CD48	103432, BioLegend, USA	1:100
Pacific blue CD150	115924, BioLegend, USA	1:200
APC CD4	100412, BioLegend, USA	1:100
APC CD8a	100711, BioLegend, USA	1:100
PE-Cy7 CD19	115520, BioLegend, USA	1:100
PE CD11b	101207, BioLegend, USA	1:100
APC-Cy7 NK1.1	156509, BioLegend, USA	1:100
APC CD86	159215, BioLegend, USA	1:100
S100A4	ab197896, Abcam, USA	1:200
Arg-1	93668S, Cell Signaling Technology, USA	1:200
iNOS	13120S, Cell Signaling Technology, USA	1:200
Alexa Fluor 488	ab150077, Abcam, USA	1:2000
F4/80	14-4801-82, Invitrogen, USA	1:200
eFluor™570, eBioscience™	41-4210-82, Invitrogen, USA	1:500
β-actin	4967, Cell Signaling Technology, USA	1:5000
STAT3	9139, Cell Signaling Technology, USA	1:2000
p-STAT3	9145, Cell Signaling Technology, USA	1:2000
IL-10	ab310329, Abcam, USA	1:200

IL-6	Ab314470, Abcam, USA	1:200

APC-Cy7 allophycocyanin-cyanine 7, *APC* allophycocyanin, *PE* phycoerythrin, *AF700* alexa fluor 700, *PE-Cy5* phycoerythrin-cyanine 5, *PE-Cy7* phycoerythrin-cyanine 7, *BV421* brilliant violet 421, *S100A4* S100 calcium-binding protein A4, *Arg-1* arginase-1, *iNOS* inducible nitric oxide synthase, *STAT3* signal transducer and activator of transcription 3, *IL* interleukin

Table S4 The primer sequences

**Gene**	**Sequence**	**Size**	**Annealing temperatures**	**GenBank accession numbers**
S100A4	F: TCCACAAATACTCAGGCAAAGAG	81	60.2	NM_001410571
	R: GCAGCTCCCTGGTCAGTAG		61.4	
β-actin	F: GCGATGCGGTTCTCTCTGG	158	62.8	NM_027886
	R: CGGCCAAGTCTTAGAGTTGTTG		61.4	
TGF-β1	F: CTCCCGTGGCTTCTAGTGC	133	62.1	NM_011577
	R: GCCTTAGTTTGGACAGGATCT		60.4	
IL-1β	F: GCAACTGTTCCTGAACTCAACT	89	60.7	NM_008361
	R: ATCTTTTGGGGTCCGTCAACT		61.4	
IL-6	F: TAGTCCTTCCTACCCCAATTTCC	76	60.8	NM_031168
	R: TTGGTCCTTAGCCACTCCTTC		61.1	
TNF-α	F: CCCTCACACTCAGATCATCTTCT	61	60.9	NM_013693
	R: GCTACGACGTGGGCTACAG		62.1	
IL-10	F: GCTCTTACTGACTGGCATGAG	105	60.2	NM_010548
	R: CGCAGCTCTAGGAGCATGTG		62.7	

S100A4 S100 calcium-binding protein A4, *TGF-β1* transforming growth factor-β1, *IL* interleukin, *TNF-α* tumor necrosis factor-α

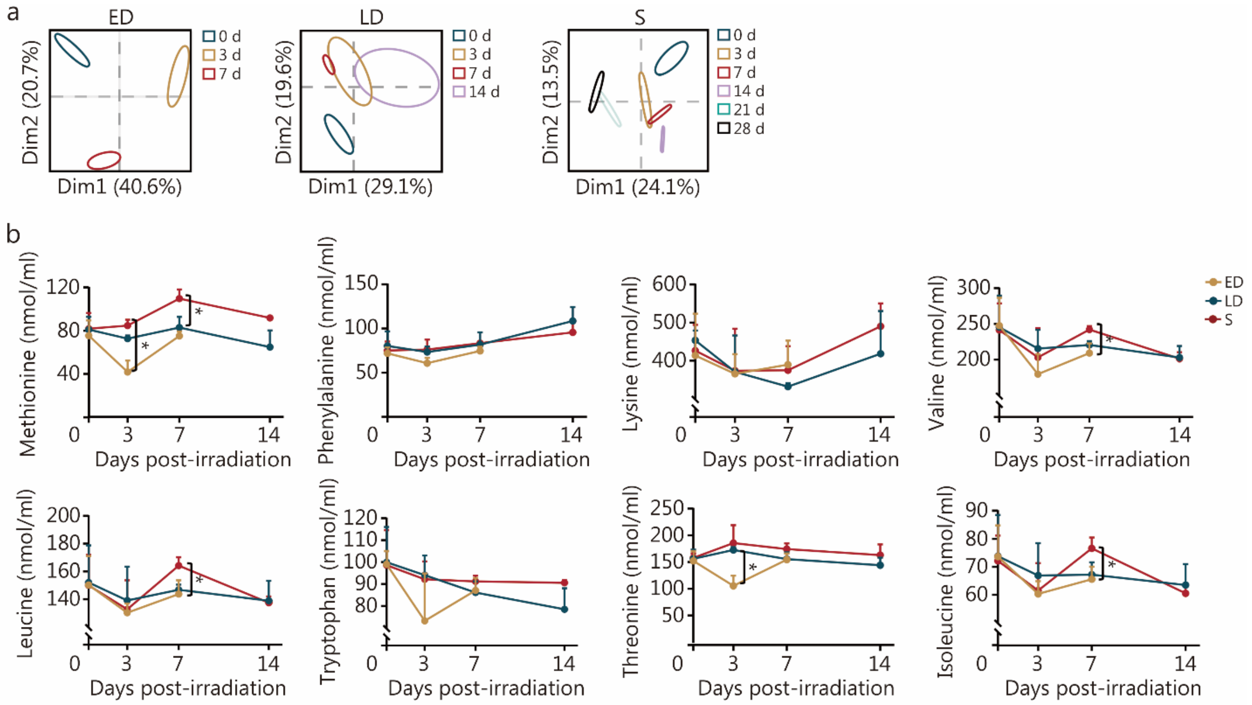

Fig. S1 The changes of amino acid contents in the serum of mice at different time points post-irradiation. **a** Principal component analysis (PCA) plot of three groups at different time points after irradiation (n = 3). **b** The changes in essential amino acids of three groups after irradiation (n = 3). The error bars indicate the standard deviation, $^{*}P < 0.05$, $^{**}P < 0.01$, $^{***}P < 0.001$ as determined Student's t-test

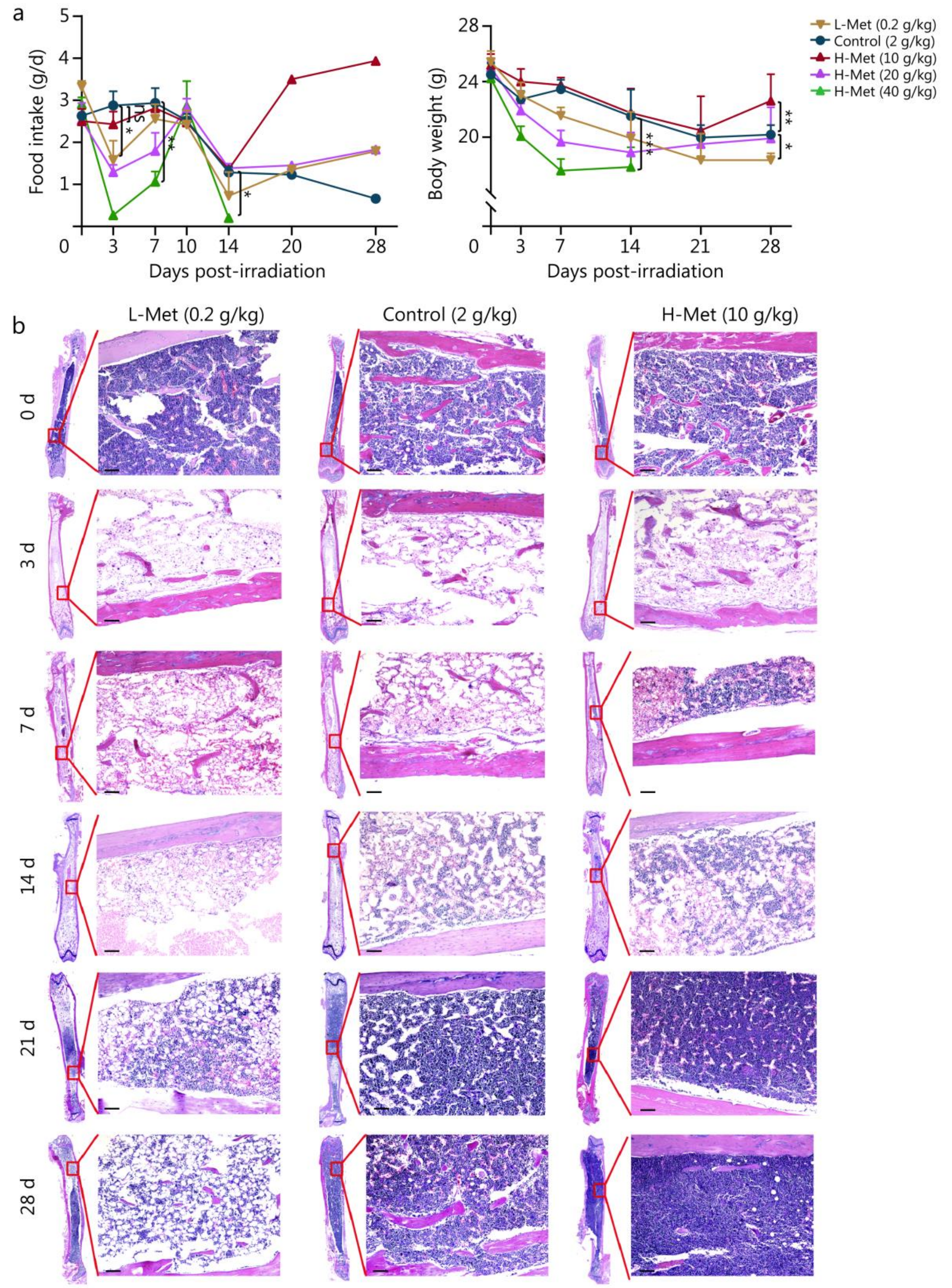

Fig. S2 Dietary methionine supplementation promotes irradiation tolerance in mice. **a** Food intake (n = 3) and body weight (n = 20) of irradiated mice fed on diets with different methionine concentrations. **b** H&E staining of femurs after irradiation with diets of different methionine concentrations. Scale bar = 100 μm. The error bars indicate the standard deviation from three or more independent experimental replicates, $^{*}P < 0.05$, $^{**}P < 0.01$, $^{***}P < 0.001$, ns non-significant, as determined by Student's t-test. L-Met low methionine diet, H-Met high methionine diet

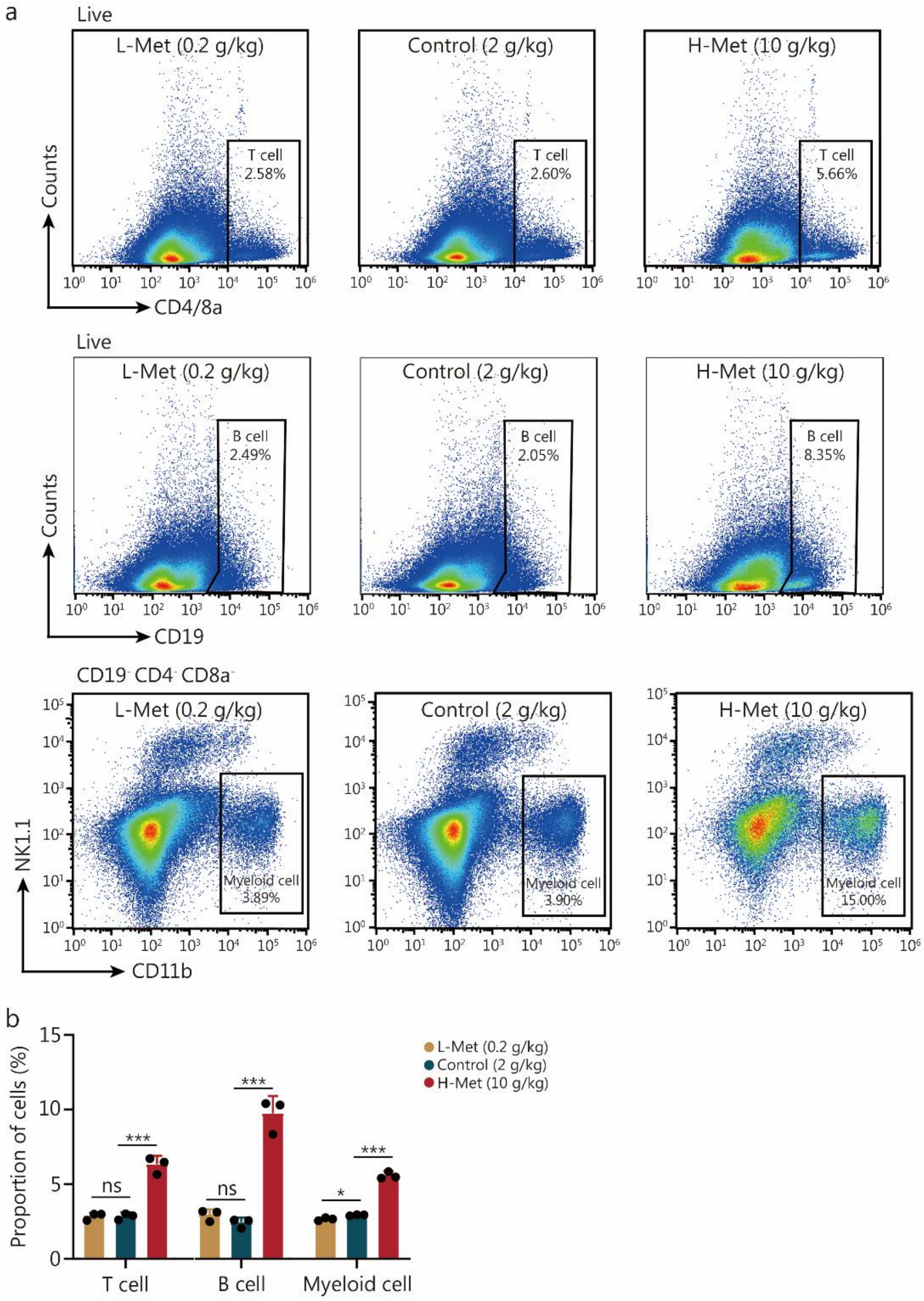

Fig. S3 Dietary methionine supplementation facilitates the recovery of bone marrow cells. **a** The proportion of B cells, T cells, and myeloid cells in bone marrow at 7 d after irradiation with different methionine diets. **b** Statistical results for T/B/myeloid cells under three independent experimental replicates ($n = 3$). The error bars indicate the standard deviation from three independent experimental replicates, $^{*}P < 0.05$, $^{***}P < 0.001$, ns non-significant, as determined by Student's *t*-test. L-Met low methionine diet, H-Met high methionine diet

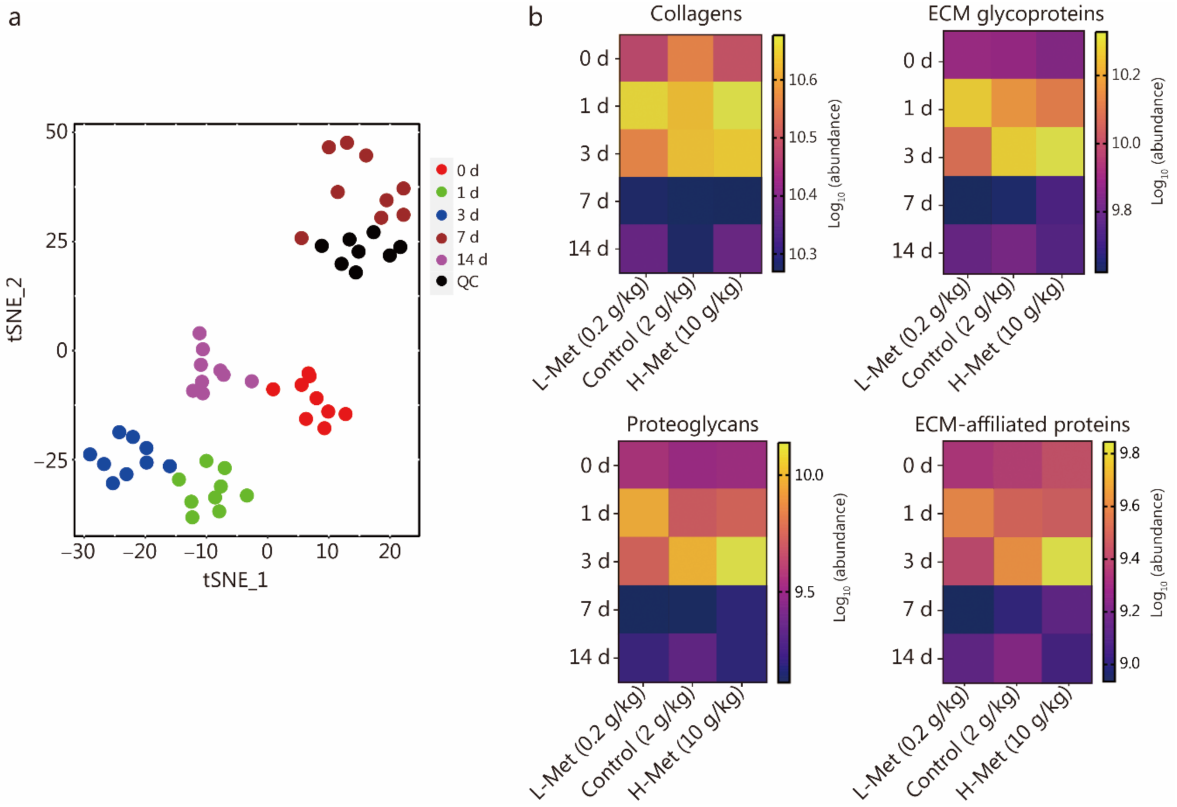

Fig. S4 The expression of bone marrow extracellular matrix (ECM) with different methionine diets after irradiation. **a** The t-distributed Stochastic Neighbor Embedding (tSNE) plot of the different groups after irradiation. **b** Heatmap of collagens, ECM glycoproteins, proteoglycans, and ECM-affiliated proteins with different methionine diets. QC quality control, L-Met low methionine diet, H-Met high methionine diet

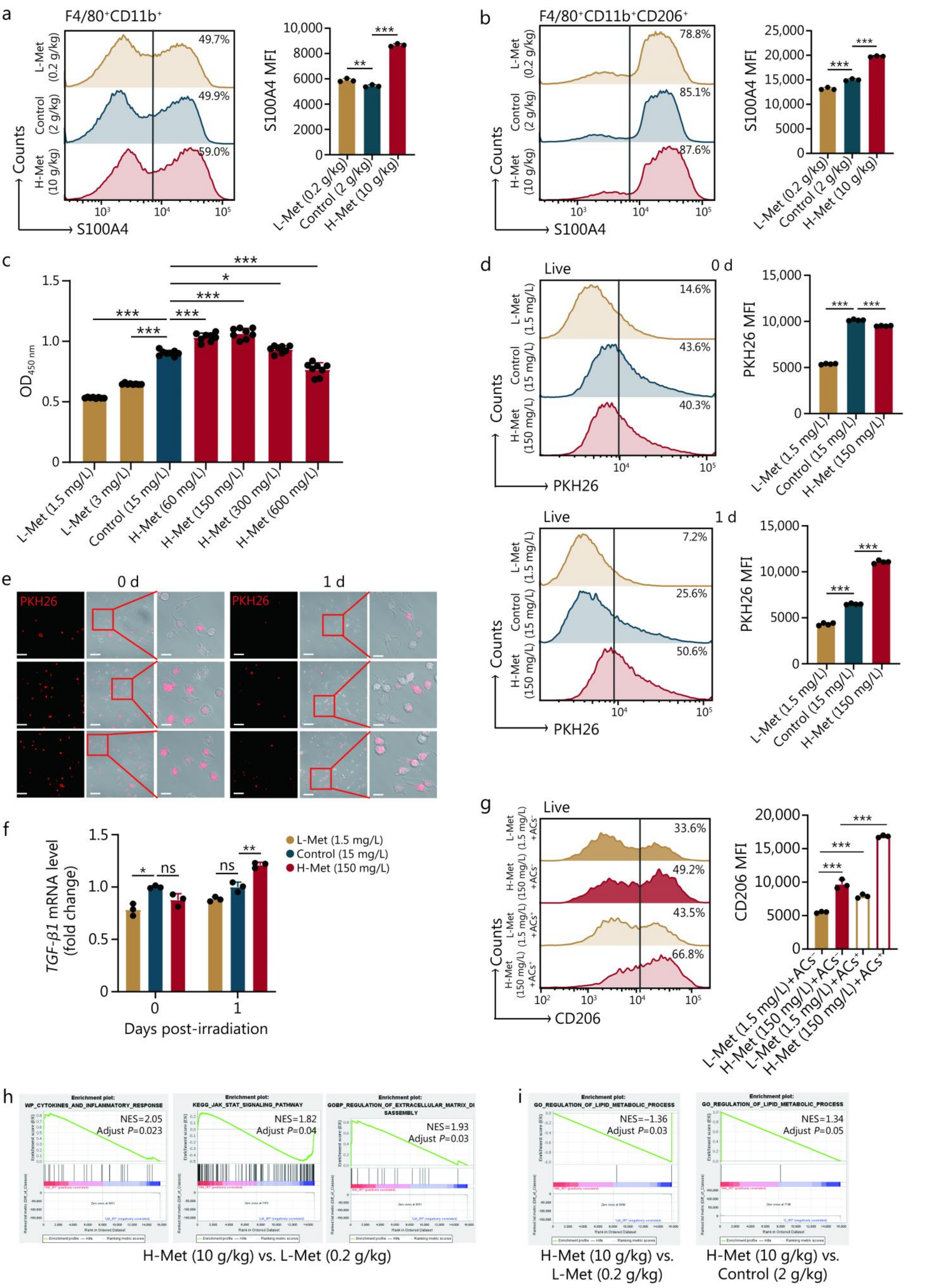

Fig. S5 The high methionine diet increased S100A4 expression in bone marrow macrophages and

promoted endocytosis. **a** S100A4 expression in macrophages at 7 d after irradiation with different methionine diets (*n* = 3). **b** S100A4 expression in CD206-positive macrophages at 7 d after irradiation with different methionine diets (*n* = 3). **c** The proliferation of BMDM with different methionine culture mediums after irradiation (*n* = 8). **d** The effect of methionine concentration on the endocytosis of BMDMs was determined by flow cytometry (*n* = 4). **e** The effect of methionine concentration on the endocytosis of BMDMs was determined by immunofluorescence. Scale bar = 10 μm. **f** The mRNA level of *TGF-β1* during the endocytosis of BMDMs with different methionine culture medium (*n* = 3). **g** The effect of methionine concentration and endocytosis on CD206 expression in BMDMs (*n* = 3). **h** Gene set enrichment analysis (GSEA) plot showed significant differences in the pathway of cytokines and inflammatory response, JAK-STAT signaling pathway, and ECM in different methionine diet groups. **i** GSEA plot showed the significant differences in lipid metabolism pathways among different methionine diet groups. The error bars indicate the standard deviation from three or more independent experimental replicates, $^{*}P < 0.05$, $^{**}P < 0.01$, $^{***}P < 0.001$, ns non-significant, as determined by Student's *t*-test. ACs^{-} BMDMs did not undergo endocytosis; ACs^{+} BMDMs did undergo endocytosis, S100A4 S100 calcium-binding protein A4, MFI mean fluorescent intensity, TGF-β1 transforming growth factor-β1, NES normalized enrichment score, L-Met low methionine diet, H-Met high methionine diet

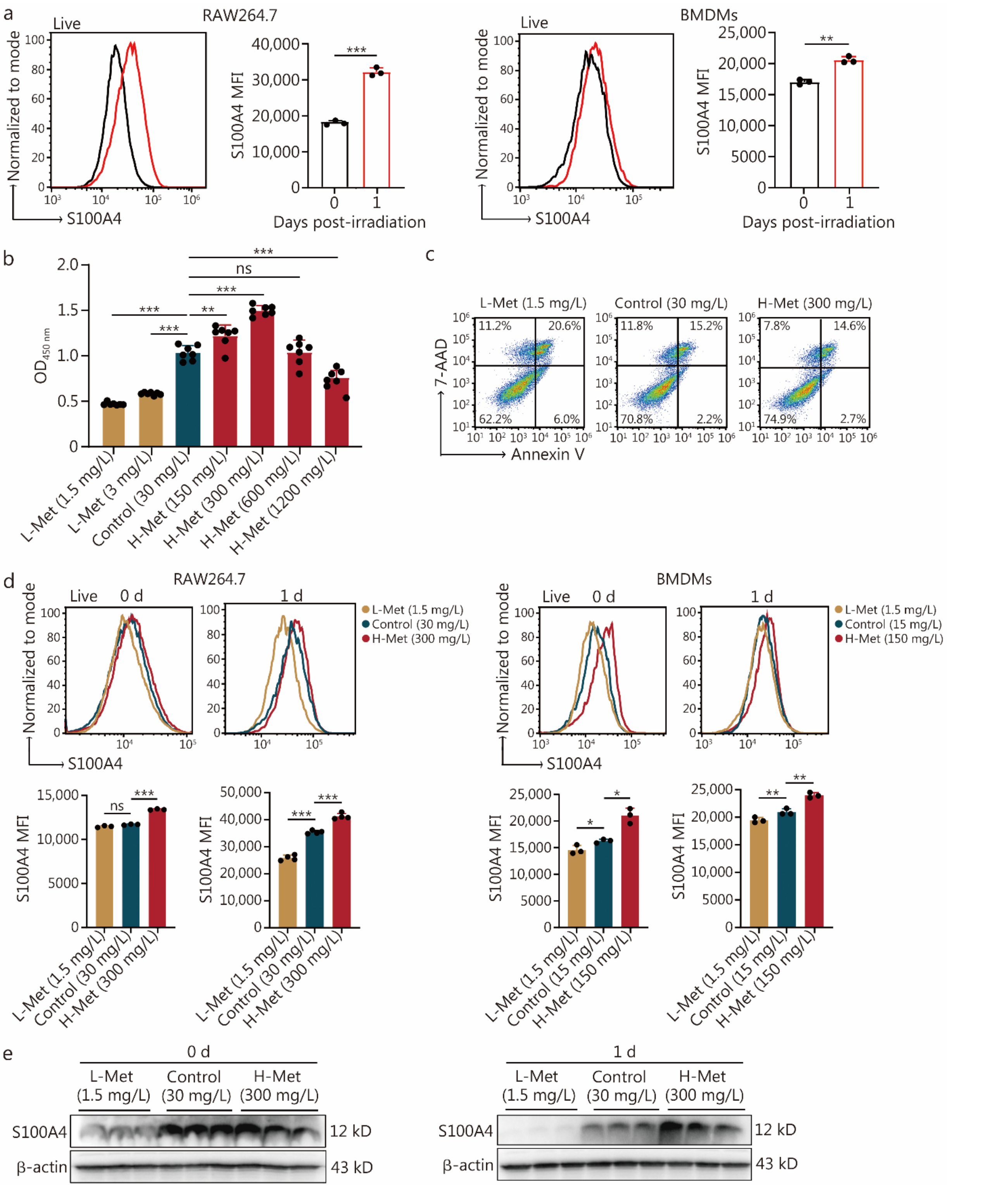

Fig. S6 The effect of RAW264.7 cells and BMDMs cultured with different methionine mediums. **a** The effect of S100A4 protein expression detected by flow cytometry in RAW264.7 cells and BMDMs at 0 d and 1 d after irradiation (n = 3). **b** The effect of cell proliferation in RAW264.7 cells with different methionine medium at 1 d after irradiation (n = 7). **c** The effect of apoptosis in RAW264.7 cells with different methionine mediums at 1 d after irradiation. **d** S100A4 protein

expression detected by flow cytometry in RAW264.7 cells and BMDMs cultured with different methionine medium at 0 d and 1 d after irradiation (n = 4). **e** S100A4 protein expression detected by Western blotting in RAW264.7 cells cultured with different methionine medium at 0 d and 1 d after irradiation. The error bars indicate the standard deviation from three or more independent experimental replicates, $^{*}P < 0.05$, $^{**}P < 0.01$, $^{***}P < 0.001$, ns non-significant, as determined by Student's t-test. S100A4 S100 calcium-binding protein A4, MFI mean fluorescent intensity, L-Met low methionine diet, H-Met high methionine diet

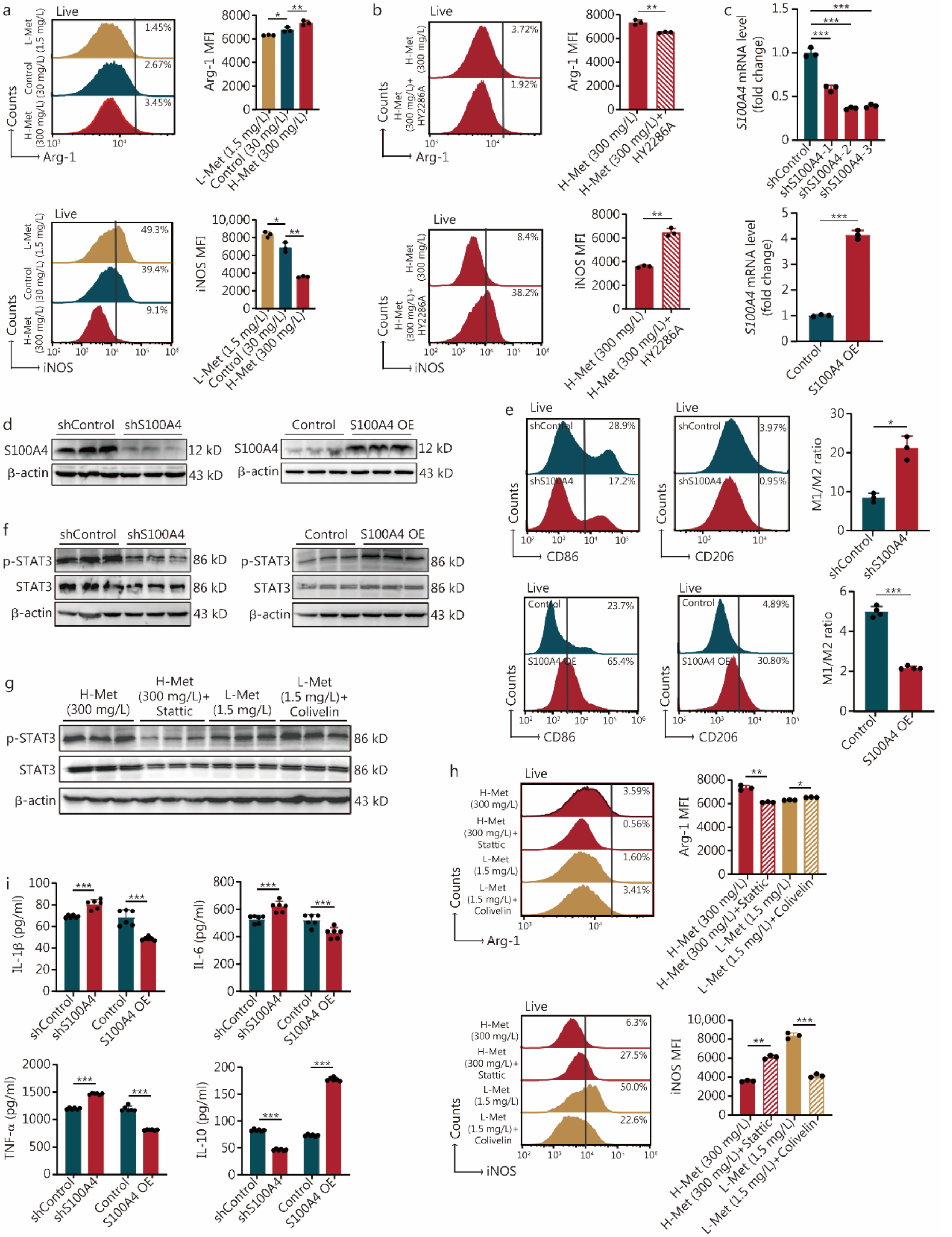

Fig. S7 S100A4 regulates macrophage polarization to participate in bone marrow inflammatory response via STAT3. **a** The expressions of Arg-1 and iNOS in RAW264.7 cells at 1 d after irradiation

with different methionine media (*n* = 3). **b** The expressions of Arg-1 and iNOS in RAW264.7 cells at 1 d after irradiation with S100A4 inhibitor (HY2286A) in 300 mg/L methionine media (*n* = 3). The efficiency of *S100A4* knockdown or *S100A4* overexpression was detected by qPCR (*n* = 3) (**c**) and Western blotting (**d**). **e** The effect of *S100A4* knockdown or overexpression on the polarization of RAW264.7 cells (*n* = 3). **f** The effect of *S100A4* knockdown or overexpression on STAT3 protein and its phosphorylation level. **g** The STAT3 protein and its phosphorylation level in RAW264.7 cells at 1 d after irradiation with STAT3 agonists (Colivelin) and inhibitors (Stattic). **h** The expressions of Arg-1 and iNOS in RAW264.7 cells at 1 d after irradiation with Colivelin and Stattic (*n* = 3). **i** The levels of inflammatory factors (IL-1β, IL-6, TNF-α, and IL-10) in the culture medium of RAW264.7 cells with *S100A4* knockdown or overexpression were detected via ELISA (*n* = 6). The error bars indicate the standard deviation from three or more independent experimental replicates, $^{*}P < 0.05$, $^{**}P < 0.01$, $^{***}P < 0.001$, as determined by Student's *t*-test. S100A4 S100 calcium-binding protein A4, shControl RAW264.7 cells were transfected with shControl, shS100A4 RAW264.7 cells were transfected with shS100A4-3, Control RAW264.7 cells were transfected with empty vector, S100A4 OE RAW264.7 cells were transfected with S100A4 overexpression vector, Arg-1 arginase 1, iNOS inducible nitric oxide synthase, MFI mean fluorescent intensity, TNF-α tumor necrosis factor-α, IL interleukin, L-Met low methionine diet, H-Met high methionine diet

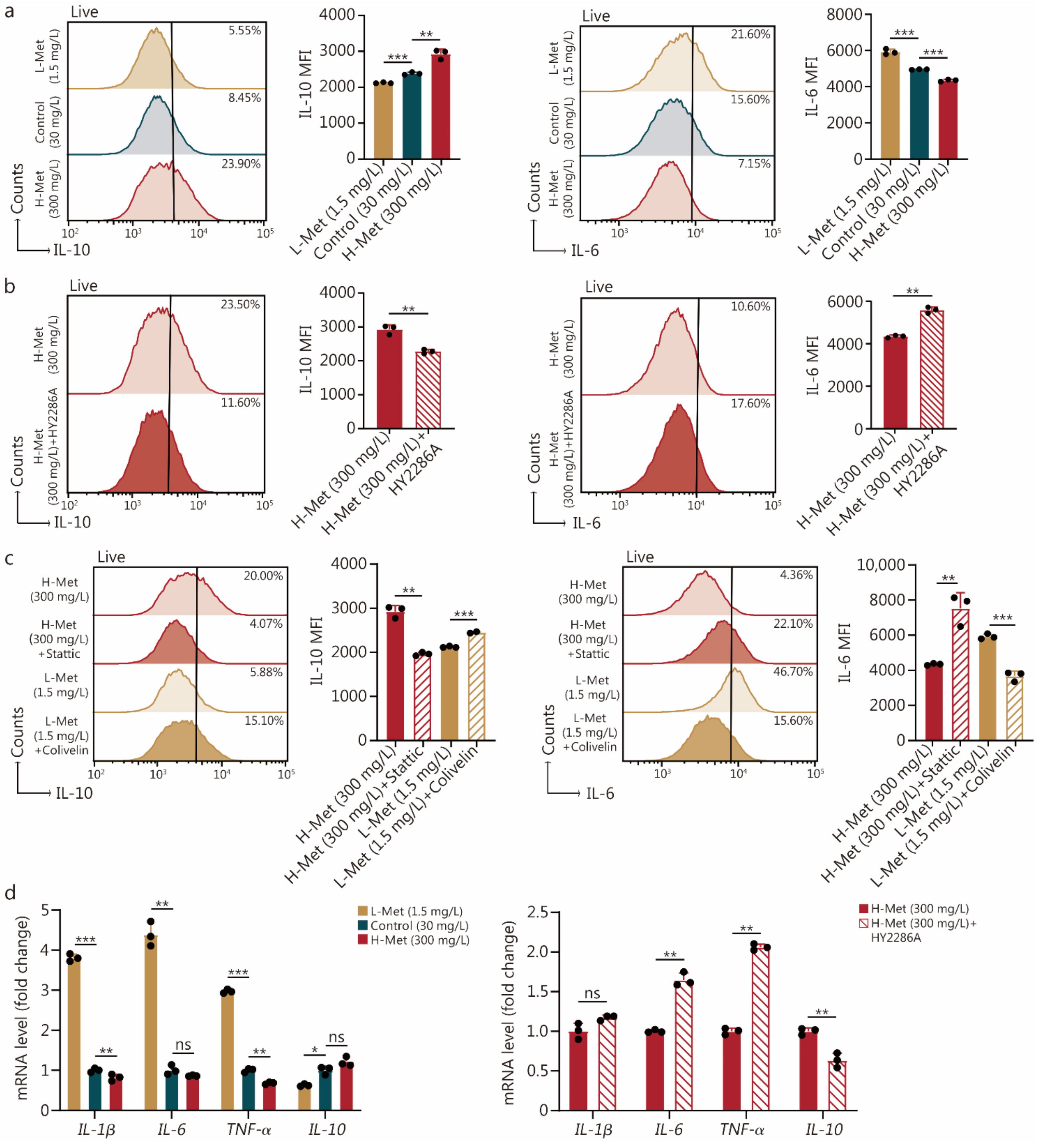

Fig. S8 The levels of inflammatory factors in RAW264.7 cells were detected via qPCR and flow cytometry. **a** The expressions of IL-10 and IL-6 in RAW264.7 cells at 1 d after irradiation with different methionine media (n = 3). **b** The expressions of IL-10 and IL-6 in RAW264.7 cells at 1 d after irradiation with S100A4 inhibitor (HY2286A) in 300 mg/L methionine media (n = 3). **c** The expressions of IL-10 and IL-6 in RAW264.7 cells at 1 d after irradiation with STAT3 agonists (Colivelin) and inhibitors (Stattic) (n = 3). **d** The levels of inflammatory factors (*IL-1β, IL-6, TNF-α*

and *IL-10*) in RAW264.7 cells were determined via qPCR (n = 3). The error bars indicate the SD from three independent experimental replicates, $^{*}P < 0.05$, $^{**}P < 0.01$, $^{***}P < 0.001$, ns non-significant, as determined by Student's *t*-test. MFI mean fluorescent intensity, TNF-α tumor necrosis factor-α, IL interleukin, L-Met low methionine diet, H-Met high methionine diet

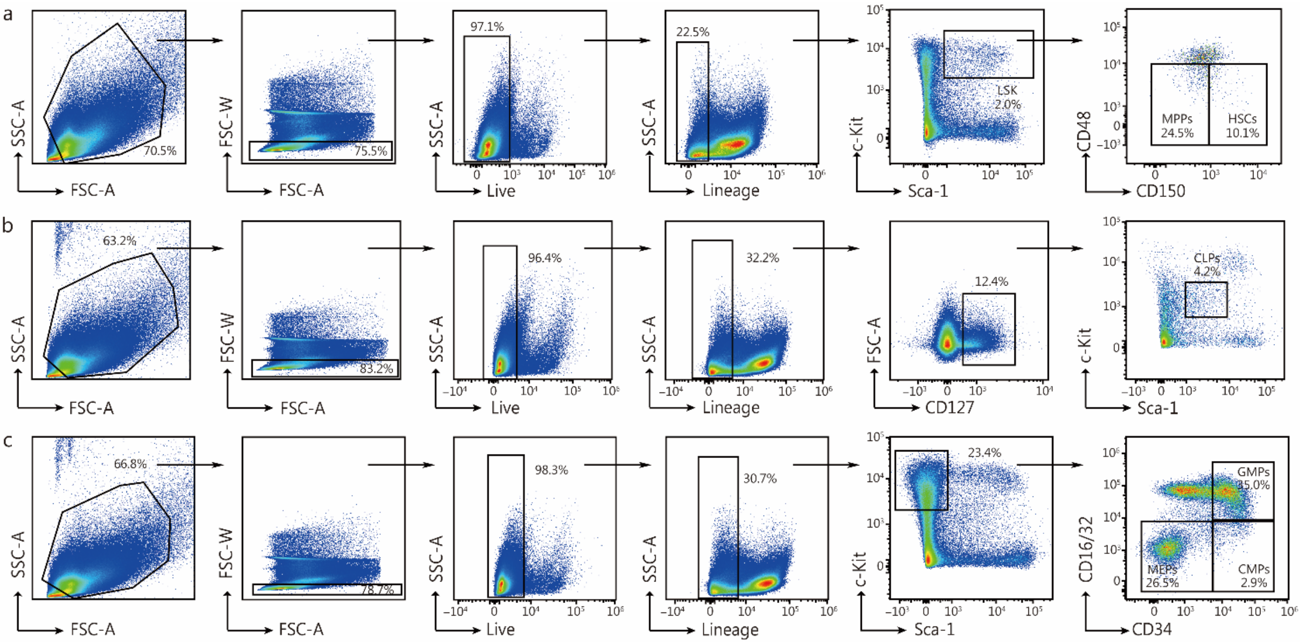

Fig S9 Flow cytometry analysis scheme for bone marrow cells. **a** The flow cytometry analysis scheme for LSK cells, HSCs, and MPPs. **b** The flow cytometry analysis scheme for CLPs. **c** The flow cytometry analysis scheme for CMPs, GMPs, and MEPs. MPPs multipotential progenitor cells, HSCs hematopoietic stem cells, CLPs common lymphoid progenitor cells, CMPs common myeloid progenitor cells, MEPs megakaryocyte-erythrocyte progenitor cells, GMPs granulocyte-macrophage progenitor cells

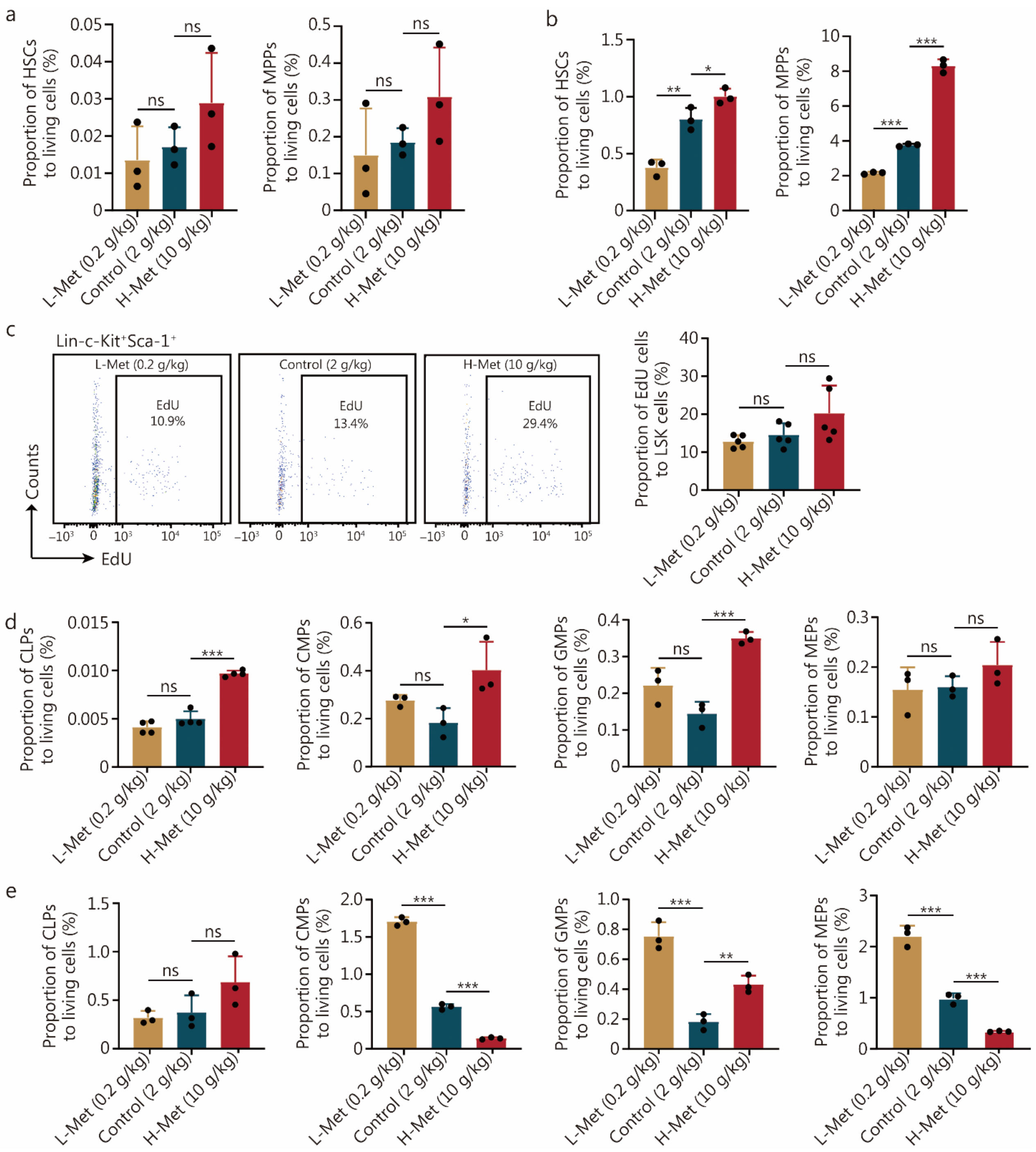

Fig. S10 Methionine promotes the proliferation and differentiation of HSC/HSPC at 7 d and 14 d post-irradiation. The proportion of MPPs and HSCs in the bone marrow of mice with different methionine diets at 7 d (**a**) and 14 d (**b**) after irradiation (n = 3). **c** The proportion of proliferating cells in LSK cells in the bone marrow of mice on different methionine diets at 7 d post-irradiation (n = 5). The proportion of CLPs, CMPs, GMPs, and MEPs in the bone marrow of mice with different methionine diets at 7 d (**d**) and 14 d (**e**) after irradiation (n = 3 – 4). The error bars indicate the

standard deviation from three or more independent experimental replicates, $^{*}P < 0.05$, $^{**}P < 0.01$, $^{***}P < 0.001$, ns non-significant, as determined by Student's *t*-test. MPPs multipotential progenitor cells, HSCs hematopoietic stem cells, CLPs common lymphoid progenitor cells, CMPs common myeloid progenitor cells, MEPs megakaryocyte-erythrocyte progenitor cells, GMPs granulocyte-macrophage progenitor cells, L-Met low methionine diet, H-Met high methionine diet
